# Cell Cycle–Dependent Differentiation Dynamics Balances Growth and Endocrine Differentiation in the Pancreas

**DOI:** 10.1371/journal.pbio.1002111

**Published:** 2015-03-18

**Authors:** Yung Hae Kim, Hjalte List Larsen, Pau Rué, Laurence A. Lemaire, Jorge Ferrer, Anne Grapin-Botton

**Affiliations:** 1 DanStem, University of Copenhagen, Copenhagen, Denmark; 2 Ecole Polytechnique Fédérale de Lausanne, Life Sciences, Institute of Bioengineering, Lausanne, Switzerland; 3 Department of Genetics, University of Cambridge, Cambridge, United Kingdom; 4 Department of Medicine, Imperial College London, London, United Kingdom; 5 Institut d'Investigacions August Pi i Sunyer, CIBER de Diabetes y Enfermedades Metabólicas Asociadas, Barcelona, Spain; Duke University Medical Center, UNITED STATES

## Abstract

Organogenesis relies on the spatiotemporal balancing of differentiation and proliferation driven by an expanding pool of progenitor cells. In the mouse pancreas, lineage tracing at the population level has shown that the expanding pancreas progenitors can initially give rise to all endocrine, ductal, and acinar cells but become bipotent by embryonic day 13.5, giving rise to endocrine cells and ductal cells. However, the dynamics of individual progenitors balancing self-renewal and lineage-specific differentiation has never been described. Using three-dimensional live imaging and in vivo clonal analysis, we reveal the contribution of individual cells to the global behaviour and demonstrate three modes of progenitor divisions: symmetric renewing, symmetric endocrinogenic, and asymmetric generating a progenitor and an endocrine progenitor. Quantitative analysis shows that the endocrine differentiation process is consistent with a simple model of cell cycle–dependent stochastic priming of progenitors to endocrine fate. The findings provide insights to define control parameters to optimize the generation of β-cells in vitro.

## Abstract 2

During the development of the pancreas, the asymmetric or symmetric differentiation of progenitor cells into endocrine cells results from stochastic priming of mitotic progenitors at different stages of the cell cycle.

## Author Summary

In order to form organs of the right size and cell composition, progenitor cells must balance their proliferation and their differentiation into functional cell types. Here we study how individual progenitor cells in the developing pancreas execute their choices to either expand their pool or differentiate into hormone-producing endocrine cells. Using live microscopy to track the genetically marked progeny of single cells, we reveal that after they divide, individual cells generate either two progenitors, two cells on the endocrine path, or one progenitor and one cell on the endocrine path. Quantitative analysis shows that endocrine differentiation is largely stochastic and that the probability of progenitor cell differentiation by the end of mid-gestation is about 20%. We propose a model in which the production of a progenitor and a differentiated cell in the pancreas results from the stochastic induction of differentiation in one daughter after cell division, rather than the unequal partitioning of molecules between two daughters at the time of division, as observed in the nervous system. Furthermore, when two daughters become endocrine cells, this results from the induction of differentiation followed by cell division—rather than two independent induction events. This model may be applicable to other organs and provides insights to optimize the generation of β-cells in vitro for diabetes therapy.

## Introduction

The pancreas is an organ performing vital exocrine and endocrine roles in nutrient metabolism and glucose homeostasis. In the mouse, multipotent pancreatic progenitor cells (MPCs) emerge from the endoderm around embryonic day 9.0 (E9.0) [1]. This population, characterized by the expression of transcription factors PDX1 (GenBank NP_032840), SOX9 (GenBank NP_035578), and HNF1B (GenBank AAH25189), eventually gives rise to all three major cell lineages of the pancreas: endocrine, acinar, and ductal [2–4]. Following early progenitor expansion, three-dimensional (3-D) organization of the pancreatic epithelium leads to the generation of an apico-basally polarized [5–7], branched tubular network. By E13.5, it exhibits its final functional compartmentalization: the distal tip domains give rise to the acinar cells of the exocrine lineage [8], whereas the SOX9^+^/HNF1B^+^ proximal trunk domain is bipotent at the population level, giving rise to the ductal and endocrine cells [3]. The endocrine lineage arises from transient NEUROG3^+^ (GenBank AAI04328.1) endocrine progenitors, as demonstrated by lineage tracing studies [2] and the absence of all pancreatic endocrine cells in *Neurog3* ^−/−^ mice [9]. NEUROG3^+^ endocrine progenitors originate from pancreatic progenitors expressing PDX1/SOX9/HNF1B during the early phases of MPC expansion and during the secondary transition spanning E12.5–15.5, with specific endocrine subtypes being specified during discrete time windows [10]. Whereas the majority of NEUROG3^+^ endocrine progenitors are post-mitotic [11] and unipotent, giving rise to only one endocrine subtype [12], we do not know whether individual PDX1/SOX9/HNF1B pancreatic progenitors give rise to both ductal and endocrine cells or are heterogeneous, encompassing cells with pre-specified lineage-restricted potentials. In this study, we ask how individual pancreas progenitors contribute to the population dynamics enabling organ expansion and endocrine differentiation.

Over the last few years cell-labelling and tracing methods have brought forth quantitative descriptions of cell differentiation. In homeostatic systems, for instance, the maintenance of a hierarchy of stem and differentiating cells can be accounted for by populations of equipotent progenitors exhibiting probabilistic fate choices [13–15]. An attempt to extrapolate these notions to developing systems has encountered some difficulties because, in these instances, the growth of the tissue needs to be taken into consideration. Notwithstanding these complications, lineage analysis of progenitor cells in the vertebrate retina indicates that, similarly to the abovementioned homeostatic systems, the distribution of clone sizes is compatible with a model in which progenitors stochastically divide in three modes: (1) symmetric self-renewing, (2) asymmetric, and (3) symmetric differentiating divisions [16–20]. Contrary to homeostatic systems, however, the probabilities of each division mode are not assumed to be fixed but to vary over time, following phases of proliferation and differentiation. These models have proven successful in explaining the distributions of clone sizes but do not explain the observed frequencies of each division type. Alternative models have been put forward that invoke deterministic asymmetric inheritance of differentiative cues at the time of division [21–24]. In general, how decisions of single cells contribute to global organ growth and differentiation in developing organs remains an open question.

Here we test some of these notions in the context of the emergence of endocrine progenitor cells from uncommitted pancreatic progenitors in the embryonic pancreas. This developmental model has a simple lineage configuration, with a reduced number of fates over well-characterized time windows, and thus provides an optimal testing framework. We use 3-D live imaging of pancreatic explants ex vivo and in vivo, together with lineage tracing at a clonal density, to address the dynamics of the progenitors of the endocrine lineage. In addition to monitoring their lineage, we determined measurements for cell cycle length, synchrony, and differentiation dynamics of these progenitors. This revealed three types of pancreatic progenitor behaviours: (1) symmetric progenitor self-renewal, (2) symmetric endocrinogenic divisions leading to two NEUROG3^+^ endocrine progenitors, and (3) asymmetric divisions generating a pancreatic progenitor and an endocrine progenitor. By live tracing individual cell fate specification events, we uncover the relationship between *Neurog3* expression timing and mitosis. We identify major differences in the onset of *Neurog3* transcription between cells stemming from symmetric and asymmetric divisions, and further show that this onset is highly synchronized between symmetrically generated sibling cells. Our analysis of such findings leads to a novel interpretation of the choice between symmetric and asymmetric cell divisions. We posit that asymmetric cell divisions are the result of the stochastic induction of endocrine fate in one of the progenitor daughters, rather than a decision made during cell division. Alternatively, if this progenitor divides a last time after induction, which is expected if the induction happens late in G1, the division will be seen as symmetric differentiative. These results argue against conventional views of asymmetric inheritance of differentiative cues at the time of division [21–24] and are instead consistent with a model of cell cycle–dependent stochastic specification of organ-specific progenitors.

## Results

### Time-Lapse Imaging Enables Pancreas Progenitor Tracking in Three Dimensions

**Fig 1:**
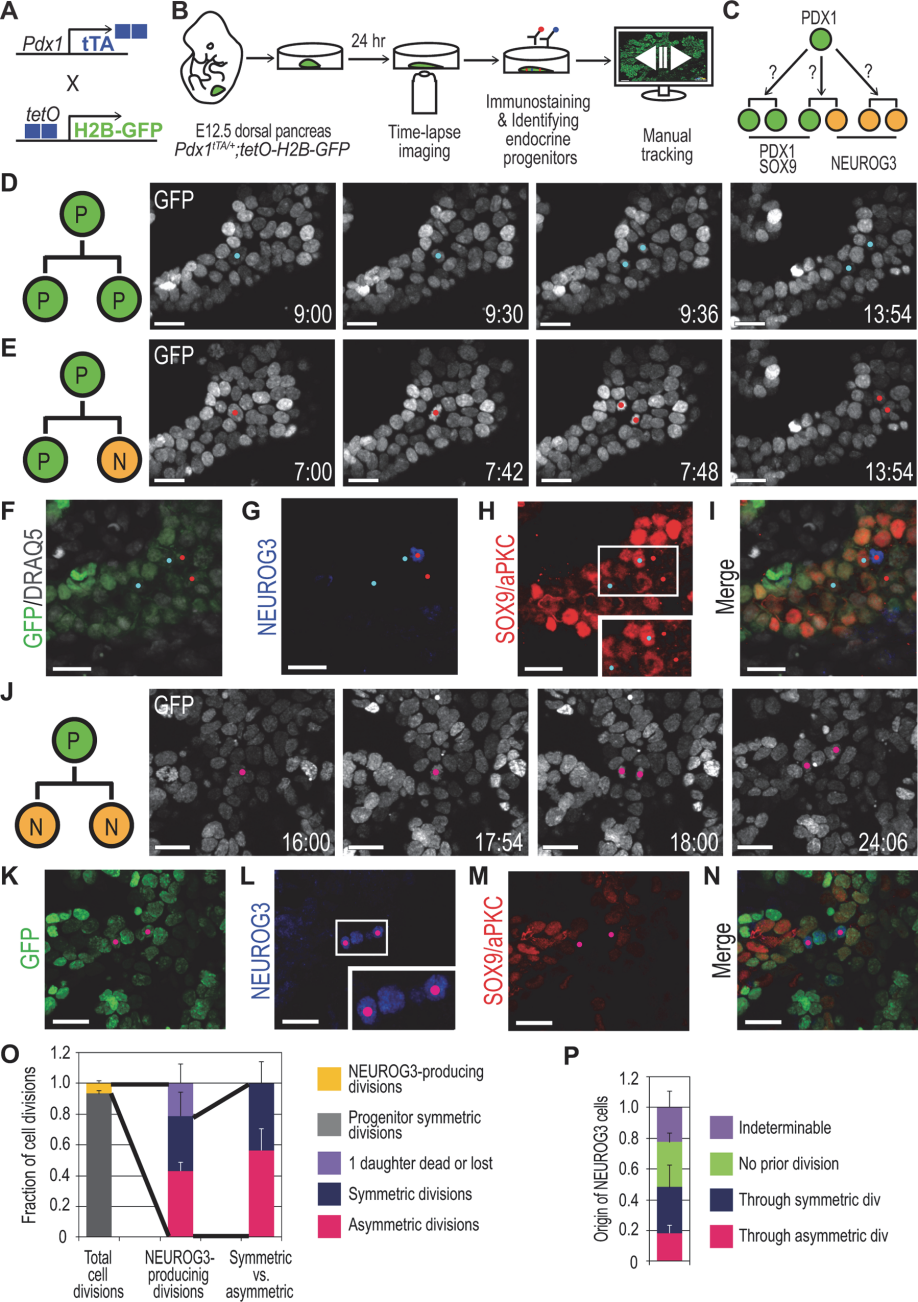
Live imaging reveals both asymmetric and symmetric emergence of NEUROG3 cells. (A) Scheme summarizing the genetic strategy to visualize PDX1^+^ pancreatic progenitors for live imaging. (B) Scheme of imaging and analysis. Pancreatic explants from E12.5 *Pdx1* ^*tTA/+*^;*tetO-H2B-GFP* embryos are cultured, and 3-D time-lapse imaging is done for 18–24 h. Then, the explants are immunostained for markers, and endocrine progenitor (NEUROG3) cells are back- and forward-tracked. (C) Model of pancreatic progenitor divisions. A PDX1^+^ progenitor can produce two PDX1^+^/SOX9^+^ progenitor daughters, two NEUROG3^+^ endocrine progenitor daughters, or one PDX1^+^/SOX9^+^ daughter and one NEUROG3^+^ daughter. (D) Still images of live imaging in 3-D maximum intensity projection from S3 Movie, illustrating a symmetric (P/P) division producing two progenitor daughters (blue spots). White nuclei correspond to H2B-GFP signal in the cells originating from the pancreas epithelium. (E) Still images from S3 Movie, illustrating an asymmetric (P/N) division producing two daughters with different fates (red spots). (F-I) Images of fixed explant with native GFP and nuclear staining DRAQ5 overlay (F) and immunostained for NEUROG3 (G) and SOX9/aPKC (H). Blue spots correspond to cells in (D) and red spots to cells in (E). Inset in (H) shows high magnification image of SOX9 staining. Note both blue spotted cells are SOX9^+^, but only one red spotted cell is SOX9^*low*^ (H), and the other red spotted cell is NEUROG3^+^ (G). (J) Still images from S4 Movie, demonstrating a symmetric (N/N) division producing two daughters with the same fate (pink spots). (K–N) Images of fixed explant with native GFP (K) and immunostained for NEUROG3 (L) and SOX9/aPKC (M). Pink spots correspond to cells in (J), and both are NEUROG3^+^/SOX9^−^. Inset in (L) shows NEUROG3 staining (four NEUROG3^+^ cells in a row) in high magnification. (O) Analysis of progenitor division patterns from live imaging. Total cell divisions are counted from four cropped positions from four live imaging movies, and fraction of NEUROG3-producing cell divisions is calculated from the corresponding positions (yellow bar over grey bar). NEUROG3-producing divisions (pink, blue, and purple bars) are counted from entire position of four movies. (P) Analysis of NEUROG3 emergence from four live imaging movies. 18.4% ± 5.0% cells emerge through P/N divisions, and 29.8% ± 14.2% through N/N divisions. 29.3% ± 5.9% do not exhibit prior division, and 22.4% ± 10.6% were either lost (17.5% ± 7.7%) or dead (8.9% ± 3.4%). Cells lost or gone out of frame were categorized as indeterminable (purple bar). Numbering denotes elapsed time in h:min, and in the cell division diagrams P indicates progenitor and N, NEUROG3 (D,E,J). Scale bars, 20 μm. Histograms and error bars represent the mean and standard deviation (*n* = 4). See S2 Table for further data.

To study how individual pancreatic progenitors contribute to pancreas expansion and to monitor their differentiation into endocrine progenitors, we conducted live imaging of explants of dorsal pancreatic buds from E12.5 *Pdx1* ^*tTA/+*^;*tetO-H2B-GFP* embryos (Fig. 1A). The buds were dissected and laid on a fibronectin-coated coverslip bottom plate, where they grew (Fig. 1B) [25,26]. After 24 h of settling time, we initiated high-magnification time-lapse live imaging in 3-D with 6-min intervals for up to 24 h. Nuclear H2B-GFP fusion protein enabled us to observe cell divisions and to track individual cell nuclei. At the end of the experiment, the explants were fixed and immunostained for markers of pancreatic progenitors (SOX9) and endocrine progenitors (NEUROG3) (S1F–I Fig.), while preserving the native green fluorescent protein (GFP) signal (S1G Fig.), which enabled us to match to the cells from the last frame of the time-lapse movies. The SOX9^+^ cells constituted the majority of GFP^+^ epithelial cells (S1I Fig.), and NEUROG3^+^ cells were mainly observed in the middle trunk region of explant (S1H Fig.) [8]. In spite of the constant exposure to laser, explants grew, showed active cell movements, apico-basal polarization, branching, acini morphogenesis, and differentiation similarly to explants that were not subjected to imaging (S1A–E,J,N,O Fig. and S1 and S2 Movies). After 18–24 h of time-lapse imaging (42–48 h post-dissection), NEUROG3^+^ cells were detected by immunostaining, showing that the differentiation process occurred ex vivo, albeit less efficiently than in vivo (S1 Table).

### Pancreatic Progenitors Divide in Three Different Modes That Differentially Contribute to Pancreas Expansion and Endocrine Differentiation

To determine how single progenitor cells contribute to balancing global pancreas expansion with endocrine progenitor generation (Fig. 1C), we systematically back-tracked NEUROG3^+^ endocrine progenitors in 3-D, as well as a random subset of SOX9^+^ pancreatic progenitors that were identified from immunostaining images and mapped onto the last frame of time-lapse movies. Once a cell division was observed through back-tracking, the sister cell was then forward-tracked, and its fate was determined by referring to the immunostaining. The tracking revealed that pancreatic progenitors divided in one of three different modes. The first type of division was symmetric, giving rise to two SOX9^+^ progenitor cells (P/P division; S3 Movie and Fig. 1D,F–I). The second type was asymmetric, giving rise to a SOX9^+^/NEUROG3^−^ pancreatic progenitor and a NEUROG3^+^ endocrine progenitor (P/N division; S3 Movie and Fig. 1E,F–I). The last type was symmetric endocrinogenic, producing two NEUROG3^+^ cells (N/N division; S4 Movie and Fig. 1J–N). In order to quantitatively account for each division mode, we analysed 1,628 divisions comprising all observed division events of *Pdx1* ^*tTA/+*^;*tetO-H2B-GFP* cells from randomly selected positions from four time-lapse movies. Thus, non-NEUROG3-producing divisions include both bi-potent progenitors and acinar cells, since Pdx1^+^ cells are multipotent at E13.5. This quantification revealed 6.6% ± 1.6% of divisions producing endocrine progenitors, and 93.4% ± 1.6% generating either progenitors or exocrine cells (Fig. 1O and S2 Table). Of all the divisions producing NEUROG3 cells that could be tracked, 56.3% ± 13.8% produced a SOX9^+^ cell and a NEUROG3^+^ cell (P/N division), and 43.7% ± 13.8% produced two NEUROG3^+^ cells (N/N division; Fig. 1O). We could determine the origin of approximately half of NEUROG3^+^ cells through P/N or N/N divisions in the past 24 h, while some NEUROG3^+^ cells either did not exhibit prior division or were either lost or dead during tracking (Fig. 1P). Cell death might be a consequence of the explant culture since apoptosis is very rare in the pancreas epithelium in vivo [27]. Taken together, these results show that at E13.5–14.5 most progenitors undergo symmetric renewing divisions, accounting for pancreas size increase, while the remaining progenitors are approximately evenly split into those undergoing symmetric endocrinogenic division and asymmetric division.

### Clonal Analysis Confirms the In Vivo Existence of Three Progenitor Division Types Based on Their Progeny

**Fig 2:**
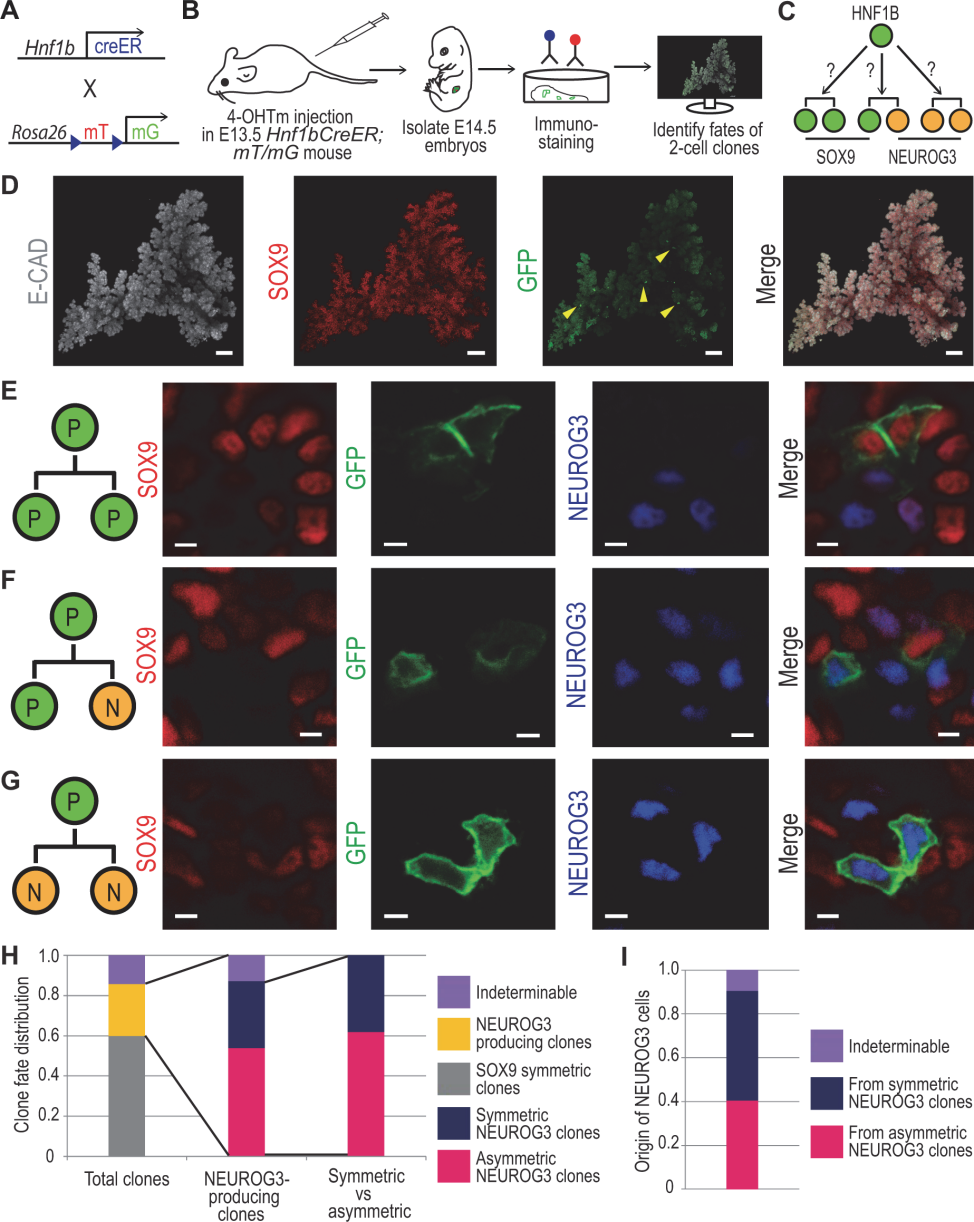
Asymmetric and symmetric divisions revealed by in vivo lineage tracing of progenitors at clonal density. (A) Scheme summarizing the genetic strategy to label HNF1B^+^ pancreatic progenitors with membrane-localized GFP reporter (mG) for lineage tracing. Upon CRE recombination, membrane-localized Tomato (mT) is excised, allowing mG expression. (B) Schematic overview of the lineage tracing strategy used to trace the fate of progeny from single progenitor cells labelled at clonal density. E13.5 pregnant mice carrying *Hnf1bCreER;mT/mG* embryos received a single intraperitoneal injection of 0.175 mg 4-OH tamoxifen. After 24 h, pancreata were subjected to whole-mount immunostaining, imaging, and 3-D reconstruction to detect recombined two-cell clones. (C) Model of two-cell clone lineage tracing. A HNF1B^+^ progenitor can produce two SOX9^+^ progenitor clones, two NEUROG3^+^ endocrine progenitor clones, or one PDX1^+^/SOX9^+^ and one NEUROG3^+^ clones. (D) Maximum intensity projection of 3-D reconstructed E14.5 dorsal pancreas after immunostaining for E-CADHERIN, SOX9, and GFP. Arrowheads indicate clones displaying recombination of the mT/mG reporter, detected by anti-GFP immunostaining, while membrane Tomato signal was diminished during staining process. (E) Optical sections from a whole-mount imaged dorsal pancreas demonstrating symmetric generation of SOX9^+^ progeny (P/P) from a single dividing progenitor cell. (F) Optical sections demonstrating clonal progeny with asymmetric fates, generating one NEUROG3^+^ and one SOX9^+^ daughter (P/N). (G) Optical sections demonstrating clonal progeny with symmetric NEUROG3^+^ fates (N/N). (H) Quantification of two-cell clone fate patterns after in vivo lineage tracing. 244 two-cell clones derived from 22 dorsal pancreata were scored according to SOX9 and NEUROG3 status. Indeterminable refers to clones that could not be categorized because of one or both daughters being both SOX9- and NEUROG3-negative after immunostaining. (I) Quantification of the number of NEUROG3^+^ cells generated by the different clone patterns. 84 NEUROG3^+^ cells were detected in 63 NEUROG3^+^ two-cell clones. Indeterminable refers to clones in which the second daughter was neither NEUROG3- nor SOX9-positive. Scale bars, 100 μm (D) and 3 μm (E–G). Histograms represent the mean (*n* = 22). See S3 Table for further data.

While ex vivo imaging enables constant monitoring of cell behaviours, it is performed in a somewhat artificial context. In order to determine whether pancreas progenitors undergo the same pattern of symmetric and asymmetric divisions in an in vivo context, we devised a clonal lineage tracing strategy (Fig. 2A) using *Hnf1bCreER* mice. Previously, this line was used to demonstrate that the E13.5 HNF1B^+^ progenitor cells give rise to ductal and endocrine cells [3]. This could be accounted for either by individual cells giving rise to endocrine and ductal cells or by heterogeneity among HNF1B^+^ cells, some giving rise to endocrine cells and others to ductal cells. To investigate this question, we subjected pregnant mice carrying E13.5 *Hnf1bCreER;mT/mG* embryos to a single low-dose intraperitoneal injection of 4-hydroxytamoxifen (Fig. 2B) to label pancreatic progenitors at a clonal density. We optimized conditions for clonal tracking leading to two-cell clones at E14.5 (Fig. 2B–G, S3 Table, and S5 Movie). Since we know from the time-lapse experiments that the majority of daughters from the same cell do not move more than 30 μm apart, we called labelled cells within 30 μm of each other a clone (S8 Fig.). Reiterations with a 60 μm radius led to similar outcomes. Whole-mount immunostaining of 22 pancreata and detection of 244 two-cell clones revealed that the majority of progenitors in which the fate could be determined divided symmetrically (P/P; Fig. 2E) into two SOX9^+^ progenitors (59.8%; Fig. 2H). This proportion is lower than the 93.4% found in the explants, in part because the cells traced by HNF1B are a subgroup of PDX1^+^ and SOX9^+^ cells traced in the explants and some of the latter will give rise to acinar cells [2,4]. In vitro lineage tracing with *Hnf1bCreER;mT/mG* explants showed that 6.3% of clones became endocrine (S2 Fig. and S4 Table). This shows that the in vivo differentiation process is more efficient than in vitro differentiation. After the 24-h tracing period, we could not yet observe any INSULIN^+^ clones in vivo, suggesting NEUROG3^−^ or SOX9^−^ clones might be in transition to endocrine differentiation. Of the NEUROG3-producing two-cell clones in which the fate of both daughters could be determined, 61.8% were asymmetric, generating one NEUROG3^+^ daughter and a SOX9^+^ progenitor (P/N; Fig. 2F,H), and the remaining were symmetric with two NEUROG3^+^ daughters (N/N; Fig. 2G,H). As a consequence, more NEUROG3^+^ cells originated from symmetric divisions (Fig. 2I). These results thus provide in vivo evidence of asymmetric and symmetric endocrinogenic progenitor divisions, as well as of symmetric renewal of progenitors, confirming the modes of divisions detected by the explant tracking data.

From the above data with regard to fate-determinable two-cell clones, we estimated expected ratios of P/P, P/N, and N/N divisions to be 69.9%, 18.6%, and 11.5%, respectively, after excluding indeterminable clones. Progenitors undergoing symmetric differentiating divisions will contribute all of their progeny to the differentiated pool, whereas asymmetrically dividing progenitors will contribute only one half of their progeny to this pool. We can therefore directly estimate the probability of differentiation of new-born cells to be 20.8% ([0.5 × 18.6] + 11.5)%, which is consistent with a net expansion of developing pancreas (S1.5 Text).

If sibling cells adopted their fate independently of each other, the expected fractions for each division type would be 62.7% for symmetric proliferative, 4.3% for symmetric differentiative, and 33% for asymmetric. Notably, these last two fractions deviate from the experimentally reported ones (Fig. 2H), contradicting the hypothesis of independent sibling fate allocation. This is further supported by statistical tests indicating a significant divergence from the independence ratios (S1.5 Text). Similar calculations can be made on the in vitro data leading to the same conclusion that a single conversion event leads to symmetric endocrine cell production (Fig. 1O and S1.5 Text).

### A New Neurog3-RFP Reporter Reveals Synchrony in Differentiation and Differentiation Delays Suggestive of Cell Cycle–Dependent Priming

**Fig 3:**
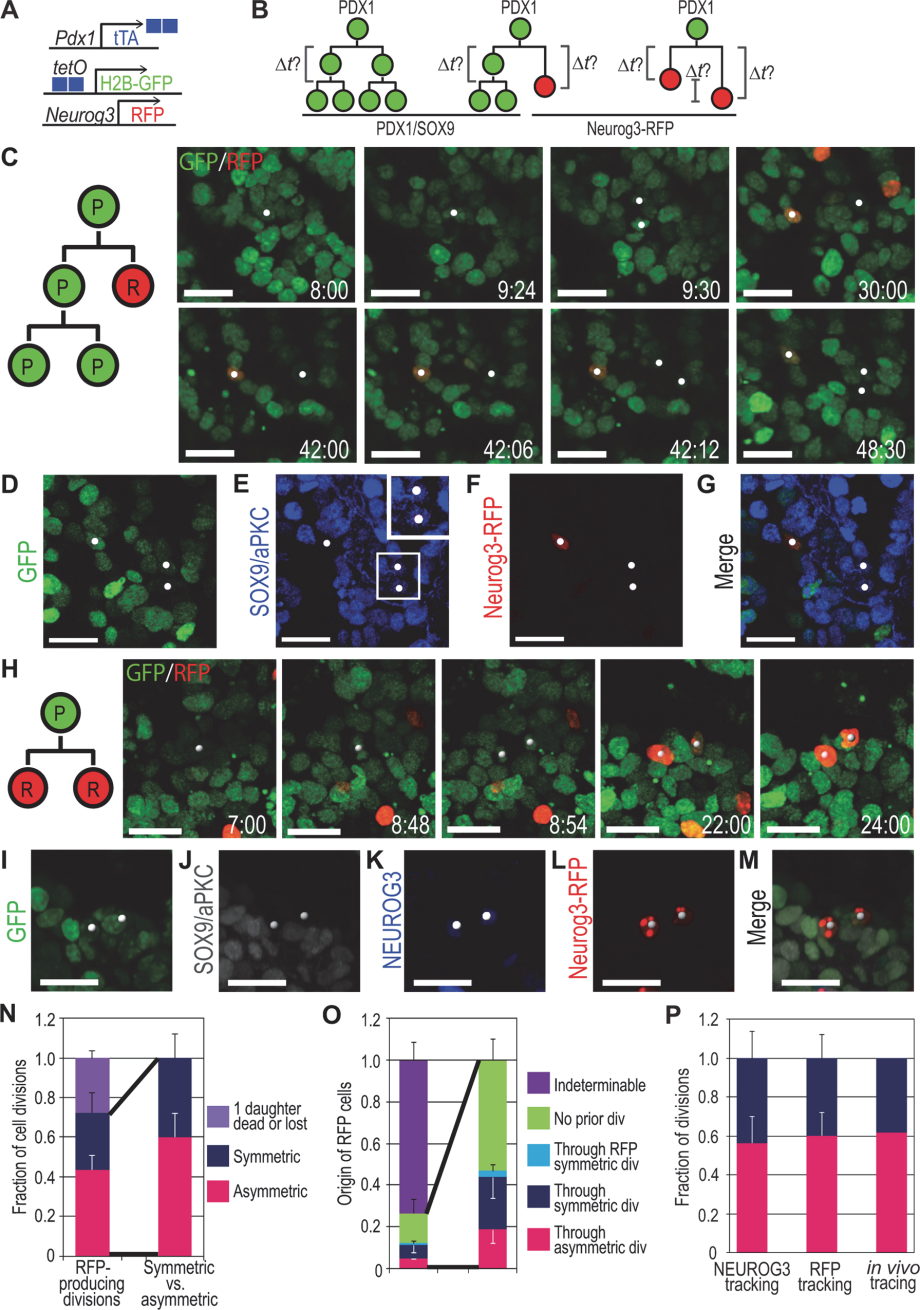
Extended live imaging with Neurog3-RFP reporter reveals the dynamics of progenitor cell cycle and differentiation. (A) Scheme summarizing the genetic strategy to visualize PDX1^+^ pancreatic progenitors and *NEUROG3* ^+^ endocrine progenitors for live imaging. (B) Model of pancreatic progenitor divisions with a *Neurog3-RFP* reporter. After second division of self-renewing progenitors, cell cycle length can be obtained. Using the *Neurog3-RFP* reporter, endocrine differentiation timing and synchrony can be obtained. (C) Still images from S6 Movie, demonstrating an asymmetric (P/R) division producing one Neurog3-RFP^+^ daughter and two other granddaughters (white spots) from a *Pdx1* ^*tTA/+*^;*tetO-H2B-GFP;Neurog3-RFP* explant. After the first division, one daughter turns on RFP (before elapsed time 30:00), and later the other daughter divides, producing two granddaughters (at elapsed time 42:12). (D-G) Images of fixed explant with native GFP (D) and immunostained for SOX9/aPKC (E) and Neurog3-RFP (F, staining for MYC-tag). White spots correspond to cells in (C), and one is RFP^+^ and two granddaughters are SOX9^+^ (E,F). Inset in (E) shows high magnification image of SOX9 staining. (H) Still images from S7 Movie, demonstrating a symmetric (R/R) division producing two Neurog3-RFP^+^ daughters (grey spots). After the division, both daughters turn on RFP. (I–M) Images of fixed explant with native GFP (I) and immunostained for SOX9/aPKC (J), NEUROG3 (K), and Neurog3-RFP (L, staining for MYC-tag). Both daughters are NEUROG3^+^/RFP^+^. (N) Fraction of RFP-producing cell divisions. Each category (pink, blue, and purple bars) was counted from three movies. (O) Analysis of RFP emergence from three live imaging movies. In three cases, RFP^+^ cells divided producing two RFP^+^ cells each (cyan bar), and the majority of RFP^+^ cells were either lost or moved out of frame during back-tracking (indeterminable, purple bar). (P) Fraction of asymmetric versus symmetric cell divisions from three different measurements: NEUROG3 tracking, Neurog3-RFP tracking, and in vivo clonal analysis. All three measurements exhibit equivalent rates of divisions. Numbering denotes elapsed time in h:min, and in the cell division diagrams P indicates progenitor and R, Neurog3-RFP (C, H). Scale bars, 20 μm. Histograms and error bars represent the mean and standard deviation (*n* = 3). See S6 Table for further data.

To investigate the dynamics of differentiation, we generated transgenic *Neurog3-RFP* reporter lines that can be used for live imaging together with H2B-GFP (Figs. 3A,B and S3A,B). Immunostaining for NEUROG3 and comparison with red fluorescent protein (RFP) from E14.5 *Neurog3-RFP* pancreas revealed that 40.1% ± 4.5% of NEUROG3^+^ cells were co-expressing RFP, while the remaining NEUROG3^+^ cells were RFP^−^ (S3C,D Fig.). Some discrepancies may be expected because of the transient nature of *Neurog3* expression and the different onset and decay kinetics of the RFP protein compared to the NEUROG3 protein (S1.3 Text). Moreover, 77.1% ± 2.8% of RFP^+^ cells were NEUROG3^−^ due to probable delay and perdurance of RFP as compared to that of NEUROG3 [28], as also seen for other reporters [29–31]. This maintenance was attested by the detection of hormones in 18.5% ± 2.3% of RFP^+^ cells. To further address the reliability of the reporter and assess its incidence in our analysis, we compared this line to the enhanced yellow fluorescent protein (EYFP) knock-add-on allele, which has been reported to show a greater overlap with NEUROG3 protein [29] and which is, in principle, less susceptible to exogenous chromatin environments, being in the endogenous locus. Our imaging of explants expressing one allele of EYFP and one of RFP (S4A,B Fig.) showed that all RFP^+^ cells were also EYFP^+^ (S4C Fig. and S5 Table), indicating no false positives due to genomic environment. Single cell tracing showed that RFP was turned on 4.7 ± 1.1 h after EYFP was detected (S4D Fig.); 11.6% ± 3.7% of EYFP^+^ cells never became RFP^+^, indicating the system was largely faithful.

**Fig 4:**
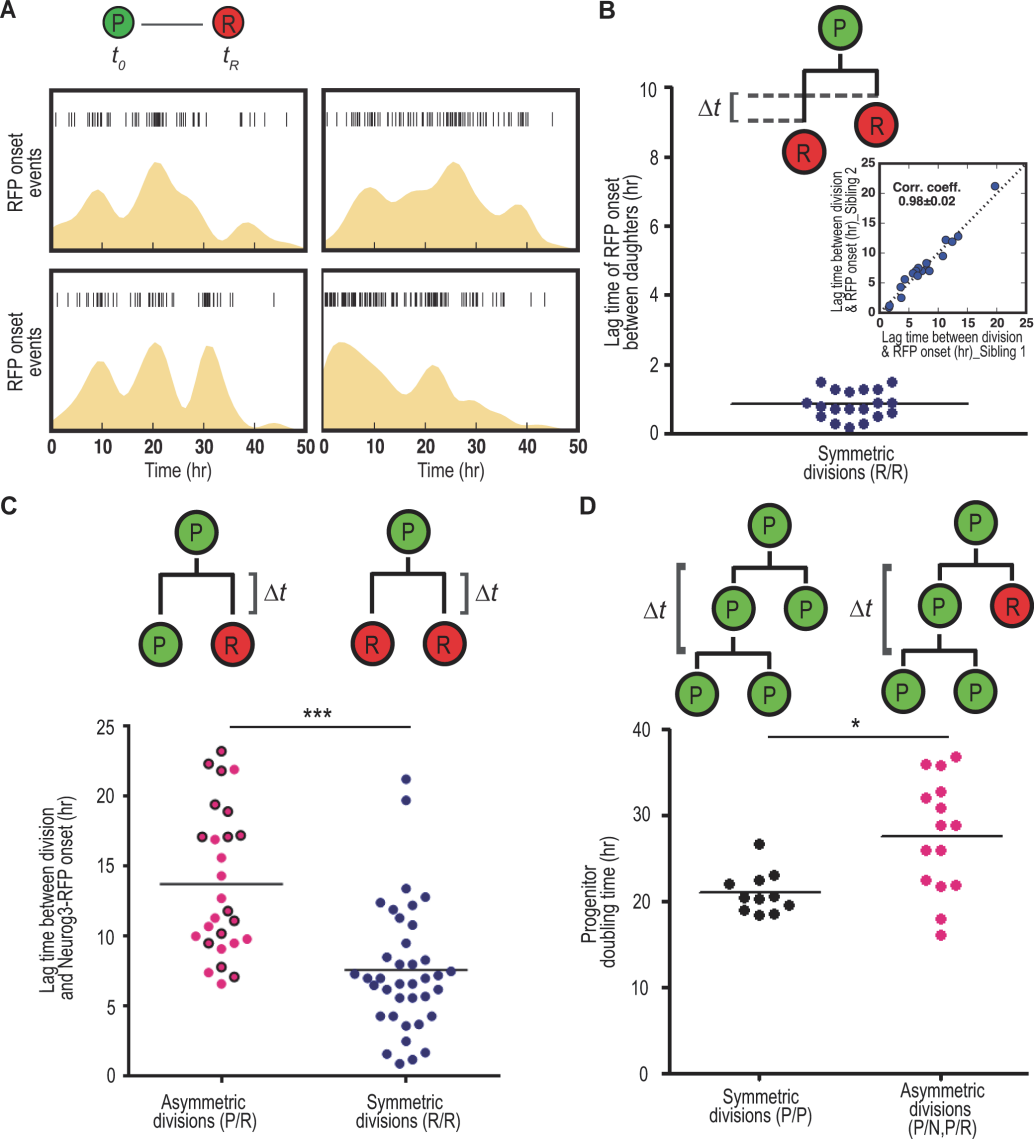
Analysis of differentiation and cell cycle dynamics from live imaging. (A) Analysis of Neurog3-RFP onset in four locations from three different time-lapse movies (*n* = 56, 89, 54, and 125, respectively). Each vertical bar symbol indicates an onset event, and the yellow area displays the probability, obtained by kernel density estimation, of an event occurring over time. These suggest that cell differentiation might not be a homogeneous process; however, further statistical analysis does not rule out this possibility (S1.4 Text). (B) Lag time of Neurog3-RFP onset between daughters derived from symmetric (R/R) divisions (*n* = 19). Symmetrically fated daughters exhibited synchronized expression of Neurog3-RFP, as pointed out by the highly correlated lag time between division and RFP onset (inset). (C) Lag time between division and Neurog3-RFP onset in asymmetric (P/R, *n* = 27) versus symmetric (R/R, *n* = 38) divisions. Note the data are pooled from three live imaging movies. RFP cells from P/R divisions took a significantly longer time to turn on RFP than cells from R/R divisions. Black-outlined red circles from P/R division (*n* = 14) indicate P/R divisions producing grand-daughters through progenitor daughter division. (D) Doubling time of progenitors originating from either symmetric (P/P, black circle) or asymmetric (P/N and P/R, pink circle) divisions. Doubling time of asymmetrically generated progenitors took longer than symmetrically generated progenitors. Statistical analyses were done using two-tailed Mann-Whitney test. *** *p* < 0.0001 and * *p* = 0.04 (C,D).

From time-lapse imaging extended to 48 h, we could observe a dynamic change of RFP signal in single cells from the onset of fluorescence: gradual increase and a subsequent decrease, which reflects the transient expression of NEUROG3 [32]. Our analysis predicts a short half-life of 5–6 h for RFP in a cell, most probably due to continuous laser exposure. We estimate a “perdurance” of detectable fluorescence of more than 20 h (see S5 Fig. and S1.3 Text) and a minimum delay between cell priming and RFP onset of approximately 5 h. Monitoring all events of RFP onset (*n* = 323; Fig. 4A) initially suggested waves of cellular differentiation at the tissue level. However, statistical analysis of the timing of onset events showed that these are also compatible with a stochastic process of cell differentiation with homogeneous differentiation rate (i.e., a Poisson process) throughout the imaging period (S6 Fig. and S1.4 Text). While we cannot rule out a periodic process underpinning *Neurog3* expression, confirmation of this would require more data points.

Similar to earlier tracking, RFP^+^ cells were back-tracked from the last time point in time-lapse movies, their prior division was monitored, and sister cells were forward-tracked. Quantifications (S6 Table) revealed that among the RFP-producing divisions where the fate of the two sisters was determinable, as follows: 60.2% ± 11.9% were asymmetric divisions producing a progenitor and a RFP^+^ daughter (P/R; S6 Movie, and Fig. 3C–G,N), and 39.8% ± 11.9% were symmetric divisions producing two RFP^+^ daughters (R/R; S7 Movie, and Fig. 3H–N). In these long time-lapse movies, many RFP^+^ cells moved out of frame or were lost due to weak GFP signal before acquiring RFP expression (Fig. 3O and S6 Table). Excluding those indeterminable RFP cells, 18.8% ± 6.6% were generated through P/R division, 25.0% ± 10.0% through R/R division, and 3.2% ± 2.8% through RFP division, while no division was seen during the movie duration for 53.0% ± 10.3% of RFP cells (Fig. 3O). These RFP tracking results thus confirmed the calculated proportions of asymmetric versus symmetric divisions established from the previous live imaging and in vivo clonal analysis (Fig. 3P).

The dynamic reporter revealed highly synchronized differentiation after divisions producing two NEUROG3 cells, the RFP signal being detected in both daughters within 0.8 ± 0.4 h of each other (Fig. 4B, correlation coefficient between lag times 0.98 ± 0.002). This outstanding synchrony confirms that it is unlikely that the two daughters are induced by independent events and suggests that mother cells have been primed to differentiate into NEUROG3 cells prior to their division. This observation further suggests a defined time between priming and NEUROG3 expression (or its RFP proxy). In addition, asymmetrically generated NEUROG3 cells exhibited a significantly longer lag time between the division and RFP onset, as shown in Fig. 4C, further supporting an interplay between cell cycle, the differentiation priming event, and the division mode.

These results on the contrasting dynamics of differentiation between cells stemming from symmetric versus asymmetric division events are obtained with the RFP reporter, for which we have established a false negative rate of 11.6% (S4C Fig.). This implies that, far from amplifying the differences between the dynamics of differentiation between the two groups, we might be underestimating them. Specifically, our reporter may miss a subset of *Neurog3*-expressing cells, thus leading to mis-allocation of around 11.6% of symmetric events to asymmetric and therefore homogenizing the two categories and reducing the differences between them (See below).

### A Model of Cell Cycle–Dependent Stochastic Priming of Progenitors Provides Quantitative Insight into the Dynamics of Cell Differentiation

**Fig 5:**
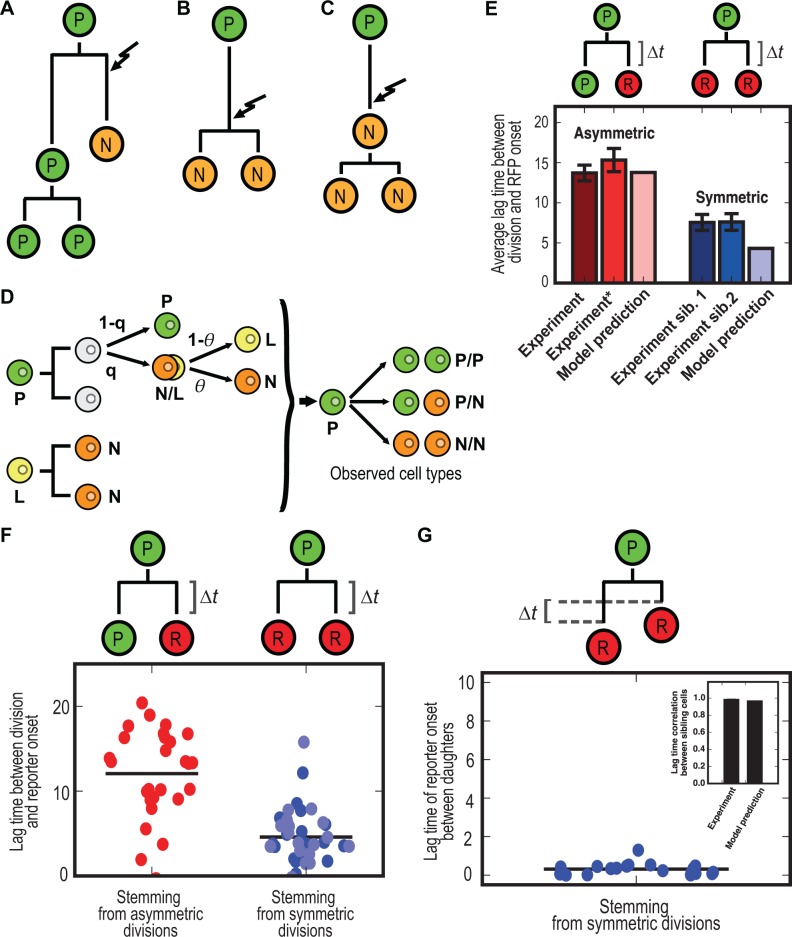
Proposed model. The observations from 3-D live imaging suggest that a distinct temporal induction of endocrine progenitor fate during the cell cycle may result in different fates of progenitors. As the majority of NEUROG3 cells are known to be largely post-mitotic [11], we propose models for three modes of cell division, resulting in NEUROG3 daughter differentiation according to a priming time point. (A) Asymmetrically fated daughter differentiation after progenitor cell division (P→P/N). Only one daughter may be induced (arrow) after the mother division, resulting in exit of cell cycle and its differentiation to NEUROG3, while the other daughter is fated as a progenitor, resulting in self-renewal. (B) Symmetrically fated daughter differentiation after progenitor cell division (P→N/N). Before mitosis, the progenitor may be induced to differentiate into an endocrine progenitor, complete the cell cycle, and divide, resulting in both daughters differentiating into NEUROG3. (C) Symmetrically fated daughters through symmetric division of endocrine progenitors (N→N/N). Considering endocrine progenitors post-mitotic, the progenitor may be induced to differentiate into endocrine progenitor, but has not yet finished the cell cycle before the cell actually differentiates into an endocrine progenitor. Therefore, to complete the cell cycle, a recently differentiated NEUROG3 cell may divide and give rise to two NEUROG3 daughters. (D–F) We have developed a mathematical model of cell cycle–dependent stochastic priming of progenitors to endocrine fate. (D) Schematic of the model in which pancreatic progenitors (P, green circles) stochastically are primed for differentiation with probability *q*. Primed cells can either exit the cell cycle and differentiate into NEUROG3 (N) with probability *θ* or conclude the cycle (L) and give rise to two NEUROG3 cells. (E) The proposed model accounts for the observed frequencies of each division mode and predicts differential RFP onset dynamics in asymmetric and symmetric divisions. “Experiment*” bar in Asymmetric category denotes asymmetric divisions accounting for a RFP daughter and a self-renewing RFP^−^ daughter (*n* = 14), whereas “Experiment” bar includes all the asymmetric divisions (refer to Fig. 4C). (F) The model with experiment-matching number of clones (out of 10,000 simulated clones) predicts a larger lag time between division and RFP onset in cells stemming from asymmetric divisions versus symmetric divisions. (G) Correlation of RFP lag times between sibling cells predicted by the model also matches that which was experimentally measured.

To try to understand the mechanism underlying the emergence of endocrine progenitors, we devised a simple mathematical model [33] of cell proliferation dynamics based on the lineage and differentiation dynamics data. The model is based on the premise that proliferating progenitor cells primed for *Neurog3*-dependent differentiation might either exit the cell cycle and become terminally differentiated or commit to complete the cell cycle and produce two terminally differentiated cells via mitosis (Fig. 5A–C). Thus in this model there are three, rather than two, cell types: (i) NEUROG3-primed (*N*) cells, which are post-mitotic; (ii) cells primed for differentiation but committed to cell cycle completion (L cells); and (iii) progenitor (*P*) cells, which will not differentiate (Fig. 5D). We assigned a probability *q* (the differentiation probability) for the differentiation of progenitors and a probability, *θ*, *for* primed cells to become N (1- *θ* to become L). Thus, the model describes the differentiation process in terms of two probabilities, which can be directly inferred from the lineage data (S1.6 Text), i.e. it has no free parameters. From the in vivo clonal analysis data, we estimate *θ* = 56.5% and, as we have already seen, *q* = 20.8%. This means that approximately one-half of the progenitors primed for differentiation become post-mitotic (P→N; 56.5%), while the other half (P→L[→N/N]; 43.5%) will undergo one last division before differentiating. Because this latter group of cells (L) is transient and contributes terminally differentiated cells (N), its expected abundance in the tissue is residuary. The model predicts that, at any given time, only 9.8% of cells in the developing tissue are primed progenitors committed to division-cycle completion (L), yet this small fraction accounts for 93.8% of the symmetric differentiative divisions (L→N/N) and might therefore explain our observation that the fates of sibling cells are linked (S1.6 Text). The remainder of symmetric differentiative divisions is interpreted to result from random, independent priming in two sister P cells.

The model also allows multiple interpretations for the probability of becoming L versus N (e.g., exposure to differentiation signals, gene expression noise, etc.). One such interpretation is the timing of the priming event after division (i.e., *θ* can be construed as accounting for a cell cycle restriction point). For instance, if a cell is primed early after division it might differentiate and halt the cell cycle, whereas if the priming event occurs late in G1, the cell might have already committed to cell cycle completion. Such specific reading of the model, which we adopt hereafter, leads to a few qualitative predictions on the dynamics of differentiation. First and foremost, the vast majority of sibling cells (93.8%) from symmetric divisions will have a perfectly synchronized differentiation program. According to the model, differentiated cells stemming from symmetric divisions shall turn on the differentiation program, on average, much earlier than those from asymmetric divisions (S1.6 Text). To quantitatively account for these predictions and compare them to the experimental data, we performed computational simulations of the model (*n* = 10,000 clones, S11 Fig.) including the observed variability in the cell cycle length as well as the dynamics of the fluorescent reporter (Figs. 5E–G, S9, S11 and S1.3, S1.4, and S1.6 Text; data deposited in the Dryad repository: http://dx.doi.org/10.5061/dryad.4b58d [34]). The simulations reproduced the differences in the onset of the reporter in cells stemming from symmetric versus asymmetric divisions (Figs. 5E,F, S6E,F, and S9) and also for the high degree of synchronization between sibling cells (Figs. 5G, S6G, and S9). Furthermore, when we included the 11.6% false negative rate in the reporter (S4C Fig.) of the model, the results were not significantly affected (S10 Fig. and S1.6 Text).

The results of the model led us to experimentally characterize the cell cycle. We used FACS-sorting of *Pdx1* ^*tTA/+*^;tetO-H2B-GFP^+^;Ngn3-RFP^−^ cells marking pancreatic progenitors at E14.5 to establish their cell cycle partition and observed that 69% were in G0/G1, 27% in S and 5% in G2/M, whereas 98% of Neurog3-RFP^+^ cells were in G0/G1 (S7 Fig.). The progenitors thus spend the majority of their time in G1. Our hypothesis is that priming in early G1 would lead to differentiation and exit of the cell cycle, and its mother would thus have an apparent asymmetric cell division. In contrast, priming in late G1 after the cell has committed to complete the cell cycle through DNA replication and mitosis would lead to an apparent symmetric differentiative cell division.

**Fig 6:**
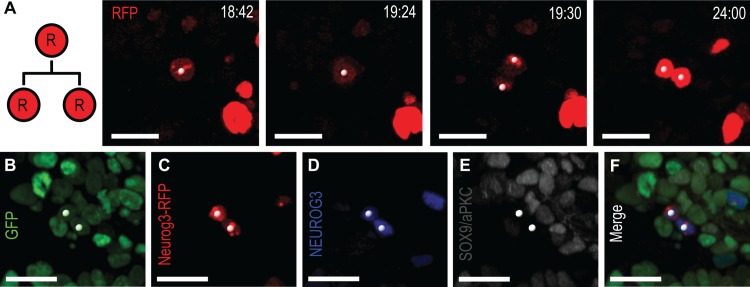
A small number of Neurog3-RFP cells divide into two NEUROG3^+^ cells. (A) Still images from S8 Movie, demonstrating division of Neurog3-RFP cell (white spots) in RFP channel from a *Pdx1* ^*tTA/+*^;*tetO-H2B-GFP;Neurog3-RFP* explant. (B–F) Images of fixed explant with native GFP (B) and immunostained for Neurog3-RFP (C, staining for Myc-tag), NEUROG3 (D) and Sox9/aPKC (E). Both daughters are NEUROG3^+^/RFP^+^. Numbering denotes elapsed time in h:min (A). Scale bars, 20 μm.

Finally, simulations also predicted the existence of a residual fraction of primed cells that would turn on the reporter immediately before dividing. Noticeably, although most RFP^+^ cells did not divide, we observed 3 cases of RFP^+^ cell division producing six cells (Fig. 6, S8 Movie, and S6 Table) and thus accounting for 3.2% ± 2.8% of all tractable RFP cells. The average time between RFP signal acquisition and division was 1.7 ± 0.8 h. This result is in agreement with the previous estimations of the progression of NEUROG3 cells through S-phase (BrdU incorporation) [11] and further shows that the NEUROG3 cells can exceptionally progress through mitosis at an early stage of their life [29].

### Analysis of the Expansion Potential of Progenitors

The longer movies enabled the observation of multiple rounds of division and the quantification of cell cycle parameters (Fig. 3B). They first confirmed that the daughter cells qualified as progenitors after asymmetric cell division based on SOX9 expression (Fig. 1H) were functionally behaving as progenitors. Indeed, among P/R divisions (*n* = 27), we observed 14 events where the RFP^−^ daughter divided, producing second-generation progeny (S6 Movie and Fig. 3C). Of those 14 cases, we observed two events where one or both granddaughters divided again, producing third-generation progeny. For all of those, immunostaining at the end point revealed that the RFP^−^ progeny were SOX9^+^ progenitors (Fig. 3E). In such cases, we could calculate the doubling time of daughter progenitor divisions, and we compared it between P/P and P/N or P/R divisions (Fig. 4D). The doubling time of self-renewing progenitors from P/P divisions was shorter than that from P/N or P/R divisions (*p* = 0.04, 21.0 ± 2.4 h and 26.5 ± 7.3 h, respectively). Moreover, the distribution of data points was greater in the asymmetric cell divisions. Finally, the time-lapse movies revealed that pancreatic epithelial cells were highly dynamic and that two daughters migrated to distances up to 64 μm apart from each other in the 24 h following division regardless of division mode (S8 Fig.).

## Discussion

In this study, we elucidate the contribution of single cell decisions to the balance between expansion and differentiation in the pancreas. Our lineage analysis, combining in vivo genetic clonal tracing with dynamic imaging in explants, reveals the existence of three kinds of divisions: symmetric progenitor self-renewal, symmetric endocrinogenic divisions leading to two NEUROG3^+^ endocrine progenitors, and asymmetric divisions generating a pancreatic progenitor and an endocrine progenitor. Furthermore, we show that progenitors are stochastically primed for endocrine differentiation, and that timing of induction in NEUROG3^+^ cells within the cell cycle establishes the division mode. Whereas late-induced cells complete the cell cycle, resulting in a differentiative symmetric division, early induced cells exit the cell cycle, in which scenario their mother would have produced asymmetric progeny. The results can alternatively be interpreted as HNF1B^+^ cells being a mix of three pre-determined populations, amongst which only one is truly bipotent. However, the clonal analysis performed in vitro shows different proportions of P/P, P/N, and N/N divisions as compared to in vivo, which would not be expected if the three HNF1B^+^ subpopulations were predetermined (unless some would preferentially die, which was not observed). Our data is most consistent with a model in which all progenitors are similar, except for their cell cycle stage, and can be primed for endocrine specification with a differentiation probability of around 20% in vivo. Future studies should reveal how this probability changes with time. For example, how it evolves to the cessation of differentiation at the end of gestation, leading to homeostatic conditions that rely primarily on slow self-duplication of differentiated populations [35]. On the other hand, a first phase of symmetric progenitor expansion followed by an increase in the probability of differentiation minimizes the time to form mature organs [36] and may also be expected to occur in the pancreas. Analogous studies are also needed in the human pancreas, as the size of the organ and the length of the differentiation stage are much greater, and several parameters such as cell cycle length of progenitors, probability of differentiation, and ratio of symmetric and asymmetric differentiative divisions may differ.

The high correlation between our in vivo and in vitro results (Fig. 3P) rules out erroneous interpretations due to in vitro artefacts and biases caused by subpopulations of progenitors marked by HNF1B at low tamoxifen doses. Spatially, the endocrinogenic divisions were observed in the centre of the pancreas where the HNF1B^+^ progenitors reside, but no areas of preferential symmetric or asymmetric division were observed.

Our dynamic data, including the synchrony in differentiation of symmetrically produced endocrine progenitor cells and their shorter lag from division to differentiation, argue that the specification event can occur at different phases in the cell cycle conditioning the ability to execute a final division or not (Fig. 5A,B). This is further supported by our analysis of the cell cycle–dependent priming model, which displays a good fit to the experimental results and provides a causal understanding of the dynamics of the process. The model proposes parameters *q* and *θ* that can be measured in other systems to test its prevalence, and our analysis of previous data in other organs suggests that it may be more general rather than specific to the pancreas [33].

Although the molecular mechanisms of *Neurog3* priming remain to be elucidated, especially whether it is under cell-intrinsic or extrinsic control, our data provide information on the general principles. Intrinsic control may be based on asymmetric inheritance of molecular components during division [21–24] or incremental or oscillatory expression of transcriptional determinants [37]. Our results strongly argue against the iterative asymmetric inheritance of differentiation cues at the time of division, as seen in *Drosophila* neurogenesis and also reported in the mouse brain [24]. Indeed, if the specification was determined at the time of division, the differentiation should occur after the same lag time in symmetric and asymmetric cell divisions. Moreover, the lag time between division and Neurog3-RFP onset is very heterogeneous ranging from 0 to 20 h (Fig. 4C), which is difficult to reconcile with a specification occurring at the time of division. If either cumulative increase or oscillations of an intrinsic determinant promoting endocrine fate lead to differentiation, the progeny of progenitor daughters arising from asymmetric division may exhibit an endocrinogenic bias. On the contrary, these progeny were all SOX9^+^ progenitors, which would rather suggest a negative bias. However, the movie duration might have been too short to observe differentiation after the second division. Moreover, we observe a slower doubling time of progenitor daughters from an asymmetric division, which may result from the inheritance of a factor that slows down the cell cycle [38–40]. Incremental specification could explain why the cell cycle time is also more heterogeneous in these progenitors. Our analyses are also compatible with extrinsic specification, for example, in the context of Notch-Delta-mediated lateral inhibition [41]. The apparent discrepancy with differentiation in the nervous system where uneven splitting of molecular cues at mitosis leads to asymmetric cell division requires further investigations in both systems. When quantified, the ratios of asymmetric and symmetric differentiation events are very similar in the pancreas and the nervous system [33], and our model would be compatible with the observation that lengthening of G1 impacts the cell division modes in the cortex [30]. Thus, an assessment of the differentiation dynamics in the nervous system similar to ours would be useful, and the possible existence of asymmetrically inherited of cues in mitotic cells in the pancreas can also be considered.

Our results reveal that the balance between expansion of progenitors and endocrine differentiation can potentially be regulated by either controlling the probability of endocrine cell induction or its timing in the cell cycle to boost the generation of endocrine cells in vitro for a cell therapy of diabetes. Our approach paves the way to establish how the frequency of division and the ratio of the different types of divisions vary over time and how their balance is controlled by signalling pathways such as Notch and FGF.

## Materials and Methods

### Mice

Genetically engineered mice used for this study were as follows: *Pdx1* ^*tTA/+*^ [42], *tetO-HIST1H2BJ/GFP* (*tetO-H2B-GFP*) [43], *Hnf1bCreER* [3], *Gt(ROSA)26Sortm4(ACTB-tdTomato*,-*EGFP)Luo/J* (*mT/mG*) [44], *N*eurog*3-EYFP* [29], and *N*eurog*3-RFP* (S3 Fig.). For embryonic stage, noon of the day when vaginal plug appeared was referred as E0.5.

The *N*eurog*3-RFP* transgenic construct (S3A Fig.) was generated by fusing 7.6 kb of the *Neurog3*-promoter [2] with a reporter construct composed of a chimeric intron; turbo RFP (Evrogen); a nuclear localization signal (NLS); a *Myc-tagC*; a bovine growth hormone polyadenylation signal (bGH-PolyA). Transgenic mouse lines were obtained by pronuclear injection of the construct (Transgenic Core Facility, EPFL, Switzerland). Two different lines were obtained initially, exhibiting similar levels of RFP signal detectable by a wide-field fluorescent microscope, and one of the lines was used for this study. All animals were handled humanely according to the authorized protocols of Switzerland and Denmark.

### 3-D Live Imaging and Cell Tracking

Dorsal pancreata from E12.5 *Pdx1* ^*tTA/+*^;*tetO-H2B-GFP* or *Pdx1* ^*tTA/+*^;*tetO-H2B-GFP*;*N*eurog*3-RFP* were cultured on a fibronectin (Sigma)-coated coverslip, adapted from the previously published protocol [25]. GFP and RFP were readily detectable under wide-field fluorescent microscopes. We used a culture medium composed of Medium 199, 10% fetal calf serum, 1% Fungizone, and 1% penicillin/streptomycin (all from Gibco). After 24 h of culture that enabled stabilization of explant flattening to approximately 80 μm thick, pancreatic explants were imaged at a single-cell resolution using Leica SP5 or SP8 confocal microscopes with a 63X glycerol immersion objective in a humidified, heated, CO_2_-controlled chamber. Tiled positions (9 [3x3] or 12 [3x4] tiles) were scanned in 256x256–280x280 format with around 40 μm Z-stack (voxel size, 0.506x0.506x1.3 μm^3^–0.880x0.880x1.25 μm^3^) every 6 min for 18–48 h. The GaAsP hybrid detection system (Leica HyD™) enabled a substantial reduction of laser power by 62.5% and increase in signal-to-noise ratio resulting in reduced scanning time, compared to conventional photomultiplier detectors. At each time point, it usually took approximately 5 min and 30 s to scan 9–12 tiled positions in 3-D. At the end point of image acquisition, the explants were fixed and prepared for whole-mount immunostaining.

Tiled images were stitched using either Leica AF6000 software or a custom-built Massive Stitcher plugin (Bioimaging and Optics Platform, EPFL, Switzerland) in Fiji. Imaris (Bitplane, Switzerland) software was used to track cells and their divisions in 3-D maximum intensity projection. Once immunostaining was done, NEUROG3^+^ endocrine progenitor cells from staining images were manually identified on the last frame of time-lapse movies with *Pdx1* ^*tTA/+*^;*tetO-H2B-GFP* explants by GFP superimposition. The identified endocrine progenitors were first back-tracked to monitor their prior divisions. Once a division was observed, the other sister was forward-tracked to the final frame, and its fate was determined from the immunostaining images. For time-lapse movies from explants with *Ngn3-RFP* in addition to *Pdx1* ^*tTA/+*^;*tetO-H2B-GFP*, RFP^+^ cells were back-tracked, and the fate of each sister was determined by immunostaining. For the quantification of total cell divisions, due to the technical difficulties in tracking all Sox9^+^ cells from the immunostaining, we did not trace all the individual cells from those 1,628 divisions, but rather subtracted the tracked divisions that produced NEUROG3 cells from the total number of divisions.

### In Vivo Clonal Analysis

Pregnant mice carrying *Hnf1bCreER;mT/mG* embryos were injected intraperitoneally with 0.175 mg 4-hydroxy (4-OH) tamoxifen (H6278, Sigma Aldrich) at E13.5. 4-OH tamoxifen was prepared as a 10 mg/mL stock in 90% corn oil and 10% ethanol and diluted to obtain the desired dose. Embryos were harvested at E14.5, and the dorsal pancreas was isolated and subjected to whole-mount immunostaining for GFP, SOX9, and NEUROG3, as indicated below. The fixation procedure eliminates native GFP and Tomato signals. After whole-mount immunostaining, dorsal pancreata were dehydrated through an ascending methanol series and subjected to clearing in a 1:2 solution of benzyl alcohol to benzyl benzoate (BABB). Cleared samples submerged in BABB were mounted on glass depression slides and imaged whole-mount using a Leica SP8 confocal microscope with a 20X oil objective at a 1024x1024 format. 3-D reconstruction of whole-mount imaged pancreata was performed using Imaris (Bitplane), enabling detection of recombined clones while preserving the spatial organization of the pancreas, thereby ensuring detection of clonal progeny by allowing interclone distance measurements. Two-cell clones were identified in 3-D space, and categorized according to SOX9 and NEUROG3 status. Recombined cells were only considered to be of clonal origin if the distance between recombined cells was less than 30 μm after the tracing period, based on live imaging data (S8 Fig.). The results were not sensitive to this parameter as using 60 μm as a maximal distance to be considered as a clone led to the same proportion of the three types of division (S2 Data).

*Hnf1bCreER*;mT/mG embryos were also used for in vitro clonal analysis by explanting pancreata at E13.5 and growing these at the air–liquid interface on 0.4 μm filters (Millipore). Explants were subjected to a 6 h pulse of 25 nM 4-OH tamoxifen in 100% ethanol to induce labelling at clonal density. Following tracing for 48 h, explants were fixed and subjected to whole-mount staining and imaging as indicated below.

### Immunohistochemistry

Whole-mount immunostaining was performed after live imaging or for pancreata harvested from the lineage tracing. After fixation with 4% paraformaldehyde (PFA) for 5 min on ice, samples were washed in phosphate buffered saline (PBS) for 5 min three times. Then, they were dehydrated through 50% and 100% methanol, and could be stored at −20°C until later use. When ready, samples were rehydrated through 50% methanol and washing solution, PBS+0.5% Triton X-100 (Tx100). Throughout the procedure, all the solutions contained 0.5% Tx100, and all the incubation was undergone in 4°C. After blocking overnight in blocking solution (1% Bovine serum albumin+0.5% Tx100), samples were incubated with primary antibodies (S7 Table) in blocking solution for 24–48 h. After washing, secondary antibodies were applied overnight, followed by washing. Alexa fluorophore conjugated secondary antibodies (Invitrogen) were used. Stained explants were kept in PBS and imaged using a confocal microscope. For quantification from explants, NEUROG3^+^ cells were counted manually, and H2B-GFP^+^ cells were counted using a custom-made macro in Fiji.

Immunostaining of frozen sections from E14.5 *Neurog3-RFP* pancreata was performed as previously described [6], and images were taken with a Leica DM5500 microscope. Quantification was obtained by manually counting immunopositive cells on every sixth section.

### Statistical Analysis and Probabilities

Statistical analyses were done by two-tailed Mann-Whitney U-test using GraphPad Prism software. Values were presented as the mean ± standard deviation.

## Supporting Information

S1 Data Excel spreadsheet containing, in separate sheets, the numerical data and statistical for Fig panels 1O, 1P, 2H, 2I, 3N, 3O, 3P, 4A, 4B, 4C, 4D, 5E, 5F, 5G, 6, and Model formulas. (XLSX)

S2 Data Excel spreadsheet containing, in separate sheets, the numerical data and statistical for Supplementary Fig panels S2, S3C, S4C, S4D, S4E, S5A, S5B, S6B, S7A, S7B, S7C, S7D, S8, and other data. (XLSX)

S1 Fig 3-D live imaging of dividing cells in pancreatic explants. (A–E) Still images of 24-h live imaging in 3-D maximum intensity projection from S1 Movie, showing an overall growth of explant and cell divisions. Numbering denotes elapsed time in h:min. (F–I) Images in 3-D projection of fixed explant with native GFP (G) and immunostained for NEUROG3 (H) and SOX9 (I). Inset shows NEUROG3^+^ cells (H, nuclear signal; arrowheads) in high magnification. Channels in (H) and (I) are masked by native GFP channel (G) to exclude non-specific background in mesenchymal regions. (J–O) Images in 3-D maximum intensity projection of immunostained control explant without imaging and laser exposure. The overall morphology after 48 h of culture, equivalent to 24-h live imaging, reveals that the epithelium branches (J). The white square is an area zoomed in (K–O). Cells in the trunk region are differentiating into NEUROG3^+^ endocrine progenitors (M, nuclear signal). The epithelium is intact as shown by E-CADHERIN staining (M), and branching ducts and acini are apically polarized as revealed by aPKC staining (O). Arrowheads in (K) indicate endocrine cell clusters. The blue channel (M) is masked by the native GFP channel (L) to exclude non-specific background in mesenchymal regions. A Z-stack is shown in S2 Movie (J–O). Scale bars, 50 μm.
(TIF)

S2 Fig NEUROG3^+^ endocrine progenitor generation is more efficient in vivo than in vitro. Comparison of two-cell clone frequency distribution from in vivo clonal analysis (left column, *n* = 244), and in vitro clonal analysis (right column, *n* = 96) using E13.5 *Hnf1bCreER;mTmG* explants cultured on filters.
(TIF)

S3 Fig *Neurog3-RFP* transgenic line. (A) Construct. A 7.6 kb *Neurog3* promoter region is linked to an intron, open reading frame of turboRFP, which contains a nuclear localization signal (NLS) and a Myc-tagC (Myc), and a bGH-PolyA signal (PolyA). The transgenic construct injection resulted in two transgenic mouse lines. (B) Optical section of a pancreatic explant from a *Pdx1* ^*tTA/+*^;*tetO-H2B-GFP;Neurog3-RFP* embryo, immunostained for NEUROG3 (blue), SOX9 (white) and aPKC (white). The RFP (red) and GFP (green) channels are native signals from each fluorescent protein. (C) Characterization of Neurog3-RFP in the E14.5 pancreas: Proportion of RFP^+^ (immunostained for Myc) (red) and RFP^−^ (white) in NEUROG3^+^ cells, and proportion of NEUROG3^+^, Hormones^+^ (identified by INSULIN [INS] and GLUCAGON [GCG]), and NEUROG3^−^/Hormones^−^ in RFP^+^ cells. (D) Optical section of E14.5 *Neurog3-RFP* pancreas, immunostained for NEUROG3 (cyan), RFP (Orange; immnostained for Myc), and INS and GCG (Magenta). White arrowheads indicate RFP^+^/NEUROG3^+^ cells, and yellow arrowheads indicate RFP^+^/NEUROG3^−^ cells. Scale bars, 20 μm. Histograms and error bars represent the mean and standard deviation (*n* = 4).
(TIF)

S4 Fig Comparison of *Neurog3-RFP* transgenic line to *Neurog3-EYFP* knock-add-on line. (A) Scheme summarizing the genetic strategy to evaluate *Neurog3-RFP* fidelity compared to *Neurog3-EYFP*. (B) Scheme of imaging and analysis. Pancreatic explants from E12.5 *Neurog3-EYFP*; *Neurog3-RFP* embryos are cultured, and 3-D time-lapse imaging is done for over 48 h. Then, EYFP- and RFP-expressing cells are tracked. (C) Quantification of EYFP and RFP cells at time 0 of time-lapse movies. Note that EYFP^+^/RFP^+^ bar (orange) includes cells that are initially RFP^−^ but acquire RFP over time, whereas EYFP^+^/RFP^−^ bar indicates cells express EYFP only throughout the movie. All RFP^+^ cells are EYFP^+^ (brown bar). Histograms and error bars represent the mean and standard deviation (*n* = 3). (D) Lag time of RFP onset after EYFP onset. RFP expression is delayed by 4.7 (± 1.1) h in EYFP^+^ cells. (E) Fluorescence intensity of EYFP and RFP in four cells in time-lapse movies. The green and red lines indicate EYFP and RFP signals, respectively. Note RFP signal is delayed by several hours, and both EYFP and RFP signals have similar trend of increase and decrease over time. See S5 Table for further data.
(TIF)

S5 Fig Dynamics of *Neurog3-RFP* during 48-hour time-lapse. (A) Fluorescence intensity of individual Neurog3-RFP cells over time. Different coloured lines indicate individual RFP cells. (B) Normalized fluorescence of all the RFP signals from (A) and aligned by 25% intensity to time 0. Black line indicates average intensity of all RFP signals. (C) Estimation of RFP half-life. Red line indicates the trend of exponential decay. RFP half-life is estimated as 5.3 h.
(TIF)

S6 Fig Analysis of Neurog3-RFP onset from time-lapse movies. (A) RFP onset time distribution from four time-lapse movie positions (*n* = 56, 89, 54, and 125). Each circle indicates onset time of RFP cell. Red, blue, and yellow circles indicate RFP cells arising from asymmetric, symmetric, and RFP divisions, respectively. (B) Analyses of RFP onset Coefficient of Variance and sliding window for oscillatory patterns. The Coefficient of Variance of each time-lapse onset distribution is equivalent to a homogeneous process, which is equal to 1.
(TIF)

S7 Fig Cell cycle analysis of e14.5 *Pdx1^tTA/+^*;*tetO-H2B-GFP;Neurog3-RFP* pancreata. (A) Flow cytometry of dissociated pancreatic cells from pooled pancreata from a litter (10 embryos: 2 GFP^+^/RFP^+^, 1 GFP^+^, 1 RFP^+^, and 6 negative pancreata) for DNA content by DAPI staining. (B) Cell cycle analysis of pancreatic progenitors (Pdx1^tTA/+^;tetO-H2B-GFP) by DAPI-stained DNA content. This panel shows 66.3% of cells in G1/G0 phase, 29.2% in S phase, and 4.5% in G2/M phase. (C) Cell cycle analysis of endocrine progenitors (Neurog3-RFP) by DAPI-stained DNA content. Note 97.2% of cells in G1/G0 phase, 1.6% in S phase and 1.2 in G2/M phase, as endocrine progenitors are mostly post-mitotic. (D) Average cell cycle of pancreatic progenitors (*n* = 3). 68.7% ± 2.1% of cells are in G0/G1, 26.6% ± 2.4% in S phase, and 4.7% ± 1.2% in G2/M phase.
(TIF)

S8 Fig Distance between two daughters relative to time after division. The *x*-axis represents the time between division and NEUROG3 end-point immunostaining. White triangles show the distance between two progenitor daughters originating from symmetric divisions (P/P), pink triangles, distance between one progenitor and one NEUROG3 cell from asymmetric divisions (P/N), and blue triangles, distance between two NEUROG3 cells from symmetric divisions (N/N). Distance from P/P divisions was measured one frame prior to daughter divisions, which indicates doubling time between mother and daughter divisions. There is therefore no data point before 18 h. Regardless of the cell division mode, the distance between daughters increased, as time after division increased (Pearson’s r, 0.81 [P/P], 0.33 [P/N], and 0.27 [N/N]). All three different modes of division showed statistically significant correlations between daughter distance and time: P/P division, *p* = 0.0025, P/N division, *p* = 0.0012, and N/N division, *p* = 0.016. The grey line indicates 30 μm threshold used for in vivo clonal analysis for two-cell clone boundaries. 90.2% of all data are under this threshold.
(TIF)

S9 Fig A model of cell cycle–dependent stochastic priming of progenitors to endocrine fate accounts for the observed RFP onset dynamics. Monte Carlo simulations of clone expansion according to the model. Each newly born cell is randomly assigned a cell cycle length from a Gamma shifted (A) with mean and variance matching the experimentally measured. (B) Distribution of times from division to the priming event for cells stemming from P and L cells. Note that in the latter the priming event occurs prior to division. (C) Distribution of times from division to the priming event for cells stemming from AD and SD divisions. (D) Distribution of set times for a reporter with constant delay. (E) Lag time between the reporter onset in sibling cells. The simulations anticipate a high degree of synchronization. (F–H) Effect of heterogeneity in the reporter delay. (F) The distribution of delays of the reporter onset is assumed Gaussian. Variability in the reporter delay has little impact in the distribution of lag times in ACD and SCD originated cells (G) and in the synchronization of sibling cells (H).
(TIF)

S10 Fig Model results including cell misclassification. In order to account for the effect of possible misclassification biases in the distribution of lag times, we performed an in silico misclassification experiment. We randomly sampled 11.6% of simulated cells stemming from symmetric divisionand classified them as stemming from asymmetric division. The resulting distribution of reporter onset lag times for this latter group features a tail at low times that might partly account for the observed (cf. Fig. 4C). This tail in the distribution, however, does not substantially affect the average lag time.
(TIF)

S11 Fig Representative in silico clone lineages generated. Temporal evolution of the expansion of five representative clones obtained with Monte-Carlo simulations of the model (*q = 0*.*2*, *θ = 0*.*55)*. *X*- axis indicates experimental time. All simulations start at time zero with one progenitor cell (P, green circle) whose cell cycle phase is set uniformly at random and run for 150 h. Progenitor cells continuously divide according to the distribution of cell cycles in S9A Fig.. Stochastic priming events are indicated by red diamonds. Events occurring early following cell division lead to cells exiting the cell cycle and differentiating (N cells, orange circles). Events taking place later on lead to primed cells that are already committed to complete the cell cycle (L cells, yellow circles) and that will give rise to two differentiated post-mitotic cells (N cells).
(TIF)

S1 Movie Time-lapse movie in 3-D maximum intensity projection of pancreatic epithelial cells. Pancreas from E12.5 *Pdx1* ^*tTA/+*^;*tetO-H2B-GFP* embryo was cultured on a fibronectin-coated coverslip plate for 24 h prior to live imaging. The explant was imaged in 3-D every 6 min in 12 positions (3x4 tiles), which were stitched after 24 h acquisition. This movie demonstrates overall expansion of explant and pancreatic progenitor divisions. Time indicates a time-lapse elapsed time in h:min:s. Frame rate: 10 frames per second (fps).
(MP4)

S2 Movie Z-stack of stained pancreatic explant after 48 h of culture. The control explant was immunostained for endocrine progenitor, NEUROG3 (blue), epithelial membrane marker, E-CADHERIN (red), and apical membrane marker, aPKC (white). Green channel is native GFP signal. Lumenized branches of epithelium are visible, and nuclear signal of NEUROG3^+^ cell are found in the epithelium. Blue channel is masked by native GFP channel to exclude non-specific back-grounds in mesenchymal region. Frame rate: 10 fps.
(AVI)

S3 Movie Time-lapse movie exhibiting symmetric (P/P) and asymmetric (P/N) pancreatic progenitor divisions. Two cell divisions are tracked in this movie (blue spots and red spots). One daughter of red spot (top; NEUROG3^+^ from immunostaining in Fig. 1G) was back-tracked from the last frame of the movie, and the other daughter was forward-tracked after the division was monitored. By referring to immunostaining at the end of the time-lapse imaging (Fig. 1), blue spotted daughters are symmetrically fated (P/P), as pancreatic progenitors (SOX9^+^), whereas red spotted daughters are asymmetrically fated (P/N), one as pancreatic progenitor (SOX9^+^) and the other as endocrine progenitor (NEUROG3^+^). *Pdx1* ^*tTA/+*^;*tetO-H2B-GFP* signal is presented in white channel. Time indicates a time-lapse elapsed time in h:min:s. Frame rate: 6 fps.
(MP4)

S4 Movie Time-lapse movie exhibiting symmetric (N/N) cell division. At the last frame, one daughter (red spot; NEUROG3^+^ from the immunostaining, Fig. 1L) was back-tracked and the other daughter was forward-tracked once the mother division was monitored. Referring back to immunostaining, the other daughter was NEUROG3^+^, as well, demonstrating symmetric endocrinogenic division (N/N). *Pdx1* ^*tTA/+*^;*tetO-H2B-GFP* signal is presented in white channel. Time indicates a time-lapse elapsed time in h:min:s. Frame rate: 6 fps.
(MP4)

S5 Movie 3-D reconstruction of whole-mount imaged *Hnf1bCreER;mT/mG* E14.5 dorsal pancreas after 24 h of in vivo lineage tracing at clonal density. Imaris software was used to perform a 3-D reconstruction of dorsal pancreas after masking of background signal in the surrounding mesenchyme. Staining for E-Cadherin (white) enables outlining of the pancreatic epithelium, while membrane GFP (green) represents labelled clones. After 360° spinning, a SOX9 symmetric two-cell clone is shown in high magnification with SOX9 in blue. Next, a NEUROG3 symmetric two-cell clone is displayed in high magnification, showing NEUROG3 in magenta. Frame rate: 15 fps.
(MP4)

S6 Movie Time-lapse movie exhibiting asymmetric (P/R) cell division from *Pdx1* ^*tTA/+*^;*tetO-H2B-GFP;Ngn3-RFP* pancreatic explant. After the mother division at elapsed time 09:30:00.000 (white spots), one daughter acquires RFP signal at 19:42:00.000, whereas the other daughter divides at 42:12:00.000. Referring to immunostaining (Fig. 3E,F), the granddaughters are progenitors (SOX9^+^), demonstrating asymmetric (P/R) division. Left panel, GFP/RFP channels and right panel, RFP channel. Time indicates h:min:s. Frame rate: 6 fps.
(MP4)

S7 Movie Time-lapse movie exhibiting symmetric (R/R) cell division from *Pdx1* ^*tTA/+*^;*tetO-H2B-GFP;Ngn3-RFP* pancreatic explant. After the mother division at elapsed time 08:54:00.000 (white spots), and one daughter turned RFP on at 18:24:00.000 and the other at 20:54:00.000. Subsequent immunostaining revealed that both of RFP^+^ daughters were still NEUROG3^+^ but SOX9^−^ (Fig. 3H). Left panel, GFP/RFP channels and right panel, RFP channel. Time indicates a time-lapse elapsed time in h:min:s. Frame rate: 10 fps.
(MP4)

S8 Movie Time-lapse movie exhibiting symmetric cell division of Ngn3-RFP cell. In rare occasions, RFP^+^ cell divides soon after acquiring RFP signal, producing two RFP^+^ daughters (white spots). Referring to immunostaining, both daughter cells are fated as NEUROG3^+^ (Fig. 6D). Left panel, RFP channel and right panel, GFP/RFP channels. Time indicates a time-lapse elapsed time in h:min:s. Frame rate: 10 fps.
(MP4)

S1 Table Fraction of NEUROG3 over *Pdx1^tTA/+^*;tetO-H2B-GFP cells from immunostaining images after time-lapse imaging. (DOCX)

S2 Table Data from NEUROG3+ cell tracking in time-lapse movies (Pdx1^tTA/+^;tetO-H2B-GFP). (DOCX)

S3 Table Data from in vivo clonal analysis (Hnf1bCreER;mT/mG). (DOCX)

S4 Table Data from in vitro clonal analysis (*Hnf1bCreER;mT/mG*). (DOCX)

S5 Table Data from Neurog3-EYFP;Neurog3-RFP explant time-lapse. (DOCX)

S6 Table Data from RFP^+^ cell tracking in time-lapse movies (*Pdx1^tTA/+^;tetO-H2B-GFP;Neurog3-RFP*). (DOCX)

S7 Table Primary antibodies used for immunostainings. (DOCX)

S1 Text Supplemental methods. (DOCX)

## Abbreviations:

3-D: 3-dimensional
4-OH: 4-hydroxy
BABB: benzyl alcohol to benzyl benzoate
bGH-PolyA: bovine growth hormone polyadenylation signal
E: embryonic day
EYFP: enhanced yellow fluorescent protein
GFP: green fluorescent protein
L: Neurog3-primed dividing cell
mG: membrane-localized green fluorescent protein
MPCs: multipotent pancreatic progenitor cells
mT: membrane-localized Tomato
N: NEUROG3 cell
NLS: nuclear localization signal
P: progenitor cell
PBS: phosphate buffered saline
PFA: paraformaldehyde
R: RFP cell
RFP: red fluorescent protein
Tx100: Triton X-100
