# Supplementary figures and images for: Cell Cycle–Dependent Differentiation Dynamics Balances Growth and Endocrine Differentiation in the Pancreas
Source: PLoS Biol. 2015 Mar 18;13(3):e1002111. doi: 10.1371/journal.pbio.1002111 (PMC4364879; doi:10.1371/journal.pbio.1002111)

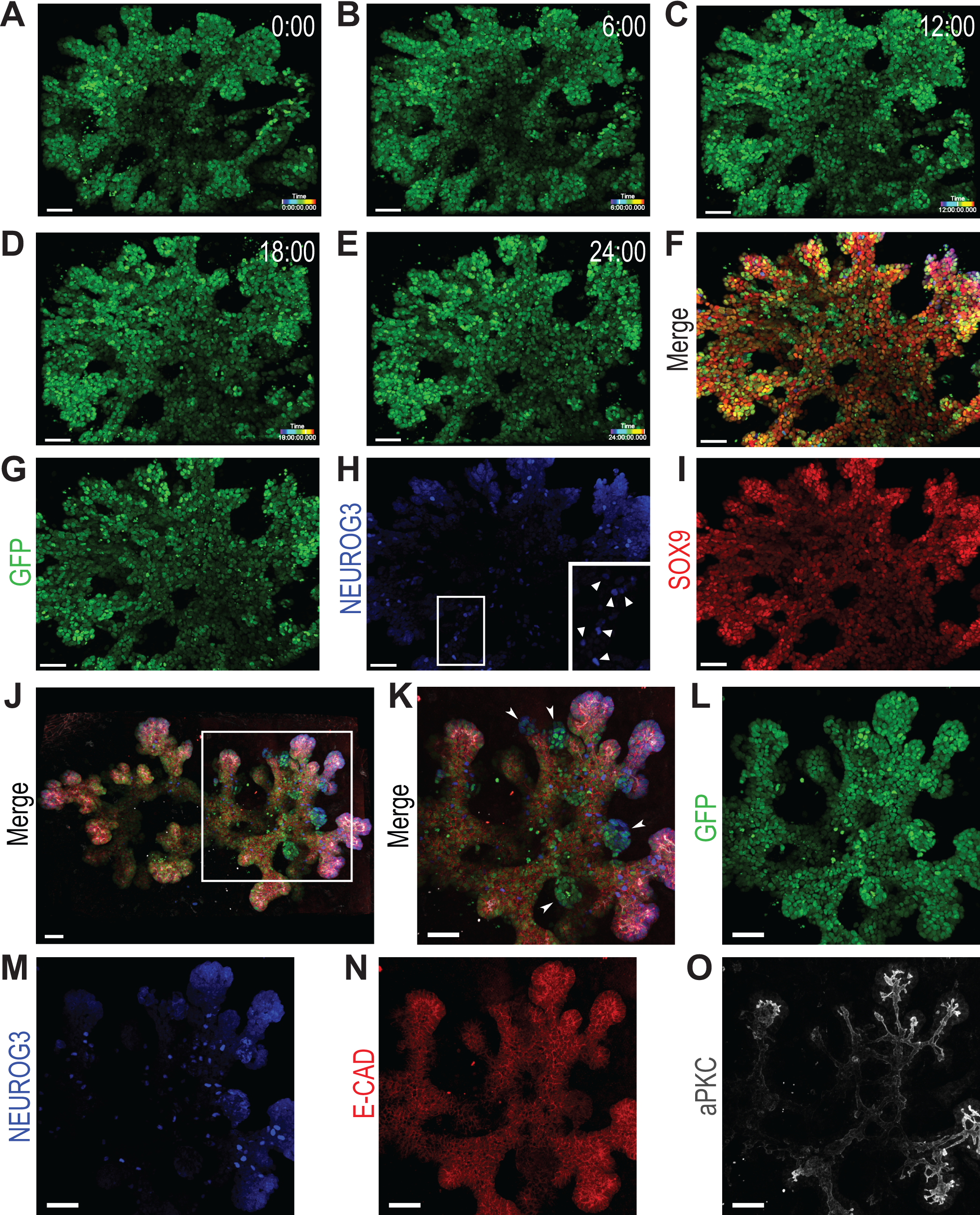

Supplement: S1 Fig — (A–E) Still images of 24-h live imaging in 3-D maximum intensity projection from S1 Movie, showing an overall growth of explant and cell divisions. Numbering denotes elapsed time in h:min. (F–I) Images in 3-D projection of fixed explant with native GFP (G) and immunostained for NEUROG3 (H) and SOX9 (I). Inset shows NEUROG3+ cells (H, nuclear signal; arrowheads) in high magnification. Channels in (H) and (I) are masked by native GFP channel (G) to exclude non-specific background in mesenchymal regions. (J–O) Images in 3-D maximum intensity projection of immunostained control explant without imaging and laser exposure. The overall morphology after 48 h of culture, equivalent to 24-h live imaging, reveals that the epithelium branches (J). The white square is an area zoomed in (K–O). Cells in the trunk region are differentiating into NEUROG3+ endocrine progenitors (M, nuclear signal). The epithelium is intact as shown by E-CADHERIN staining (M), and branching ducts and acini are apically polarized as revealed by aPKC staining (O). Arrowheads in (K) indicate endocrine cell clusters. The blue channel (M) is masked by the native GFP channel (L) to exclude non-specific background in mesenchymal regions. A Z-stack is shown in S2 Movie (J–O). Scale bars, 50 μm. (TIF) [file pbio.1002111.s003.tif]

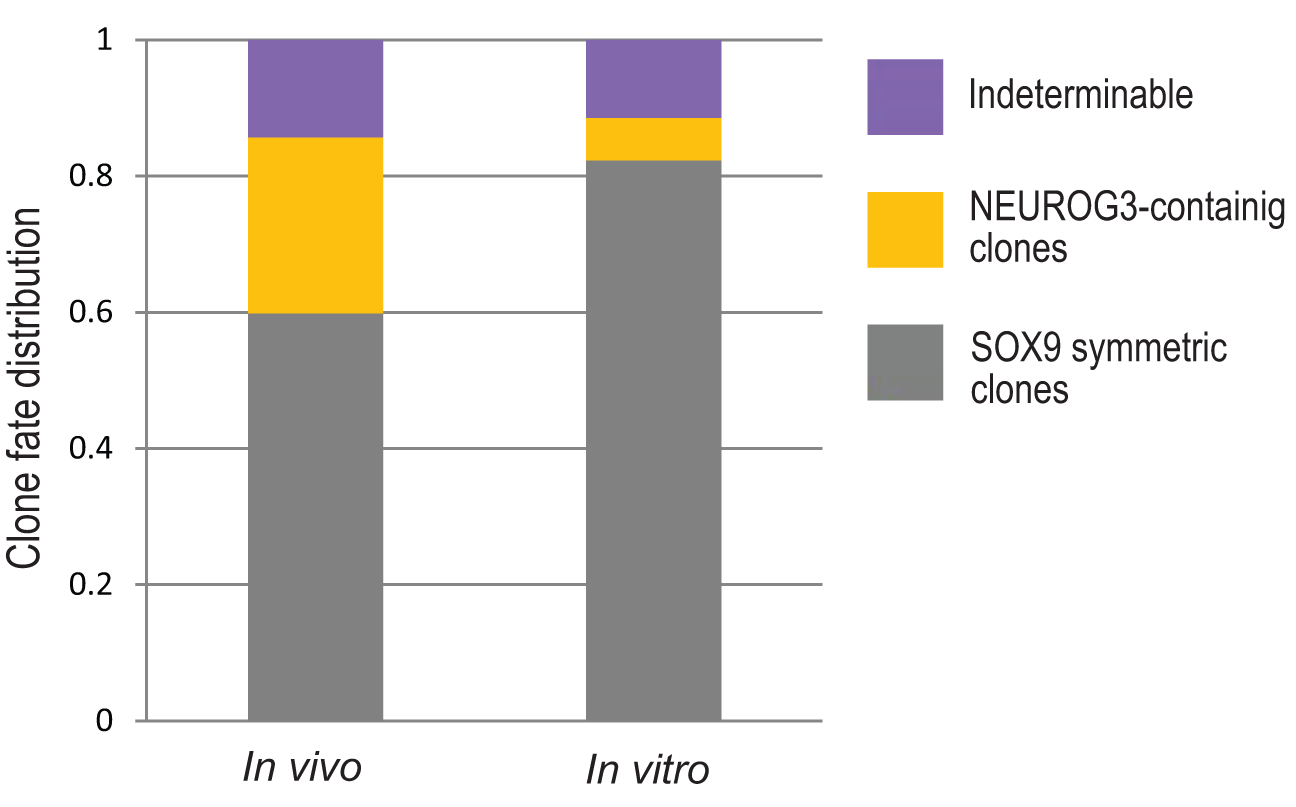

Supplement: S2 Fig — Comparison of two-cell clone frequency distribution from in vivo clonal analysis (left column, n = 244), and in vitro clonal analysis (right column, n = 96) using E13.5 Hnf1bCreER;mTmG explants cultured on filters. (TIF) [file pbio.1002111.s004.tif]

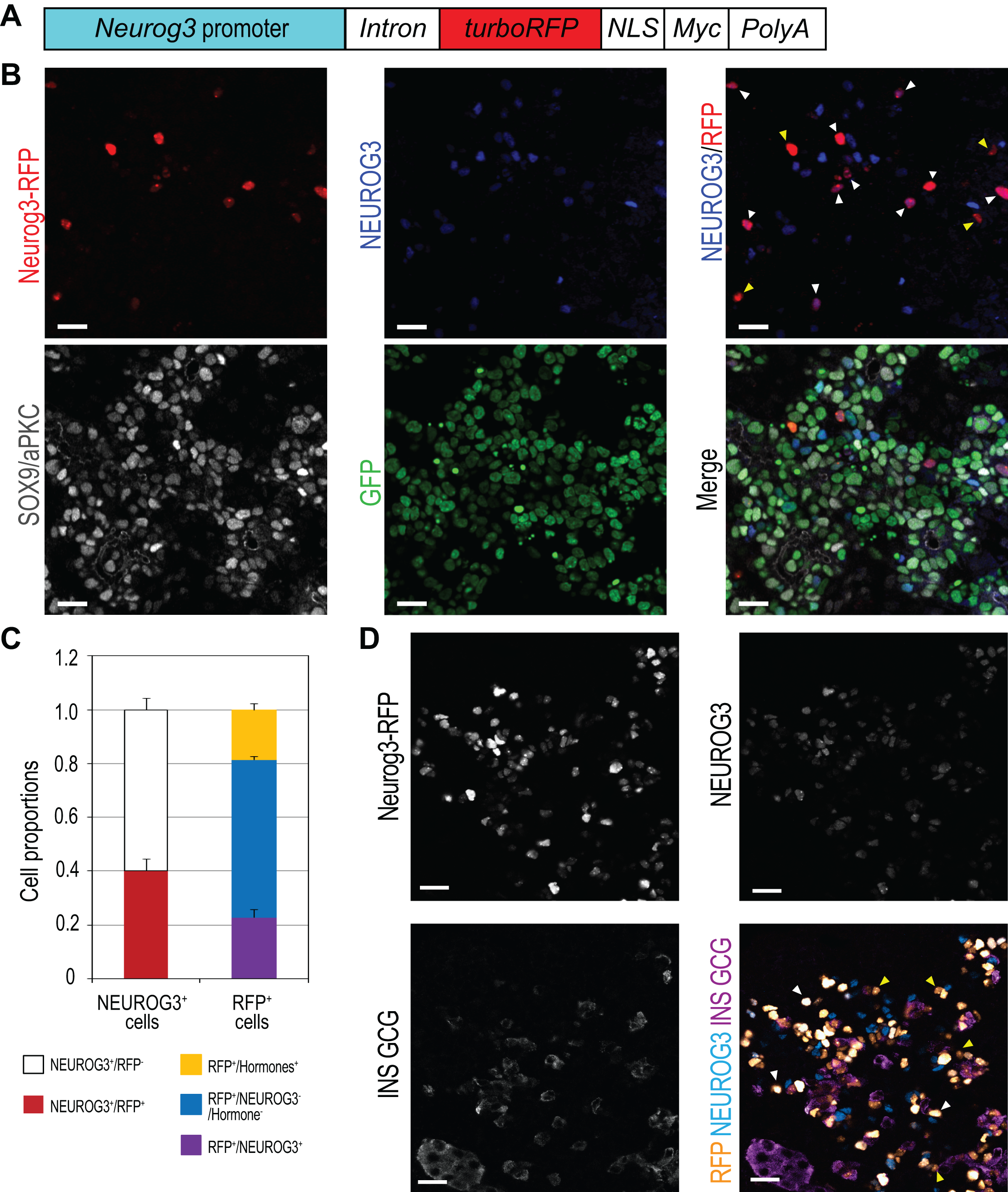

Supplement: S3 Fig — (A) Construct. A 7.6 kb Neurog3 promoter region is linked to an intron, open reading frame of turboRFP, which contains a nuclear localization signal (NLS) and a Myc-tagC (Myc), and a bGH-PolyA signal (PolyA). The transgenic construct injection resulted in two transgenic mouse lines. (B) Optical section of a pancreatic explant from a Pdx1 tTA/+;tetO-H2B-GFP;Neurog3-RFP embryo, immunostained for NEUROG3 (blue), SOX9 (white) and aPKC (white). The RFP (red) and GFP (green) channels are native signals from each fluorescent protein. (C) Characterization of Neurog3-RFP in the E14.5 pancreas: Proportion of RFP+ (immunostained for Myc) (red) and RFP− (white) in NEUROG3+ cells, and proportion of NEUROG3+, Hormones+ (identified by INSULIN [INS] and GLUCAGON [GCG]), and NEUROG3−/Hormones− in RFP+ cells. (D) Optical section of E14.5 Neurog3-RFP pancreas, immunostained for NEUROG3 (cyan), RFP (Orange; immnostained for Myc), and INS and GCG (Magenta). White arrowheads indicate RFP+/NEUROG3+ cells, and yellow arrowheads indicate RFP+/NEUROG3− cells. Scale bars, 20 μm. Histograms and error bars represent the mean and standard deviation (n = 4). (TIF) [file pbio.1002111.s005.tif]

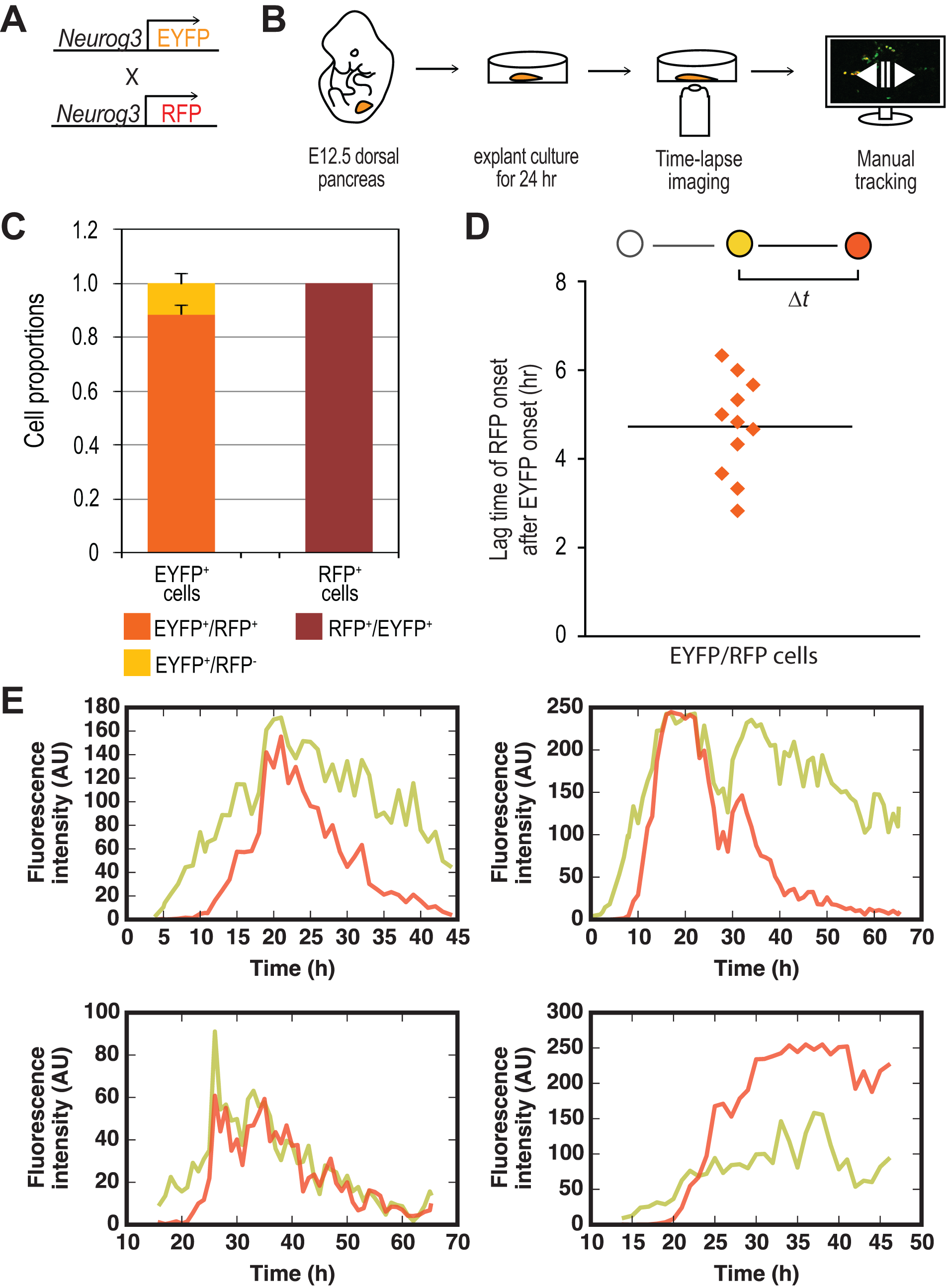

Supplement: S4 Fig — (A) Scheme summarizing the genetic strategy to evaluate Neurog3-RFP fidelity compared to Neurog3-EYFP. (B) Scheme of imaging and analysis. Pancreatic explants from E12.5 Neurog3-EYFP; Neurog3-RFP embryos are cultured, and 3-D time-lapse imaging is done for over 48 h. Then, EYFP- and RFP-expressing cells are tracked. (C) Quantification of EYFP and RFP cells at time 0 of time-lapse movies. Note that EYFP+/RFP+ bar (orange) includes cells that are initially RFP− but acquire RFP over time, whereas EYFP+/RFP− bar indicates cells express EYFP only throughout the movie. All RFP+ cells are EYFP+ (brown bar). Histograms and error bars represent the mean and standard deviation (n = 3). (D) Lag time of RFP onset after EYFP onset. RFP expression is delayed by 4.7 (± 1.1) h in EYFP+ cells. (E) Fluorescence intensity of EYFP and RFP in four cells in time-lapse movies. The green and red lines indicate EYFP and RFP signals, respectively. Note RFP signal is delayed by several hours, and both EYFP and RFP signals have similar trend of increase and decrease over time. See S5 Table for further data. (TIF) [file pbio.1002111.s006.tif]

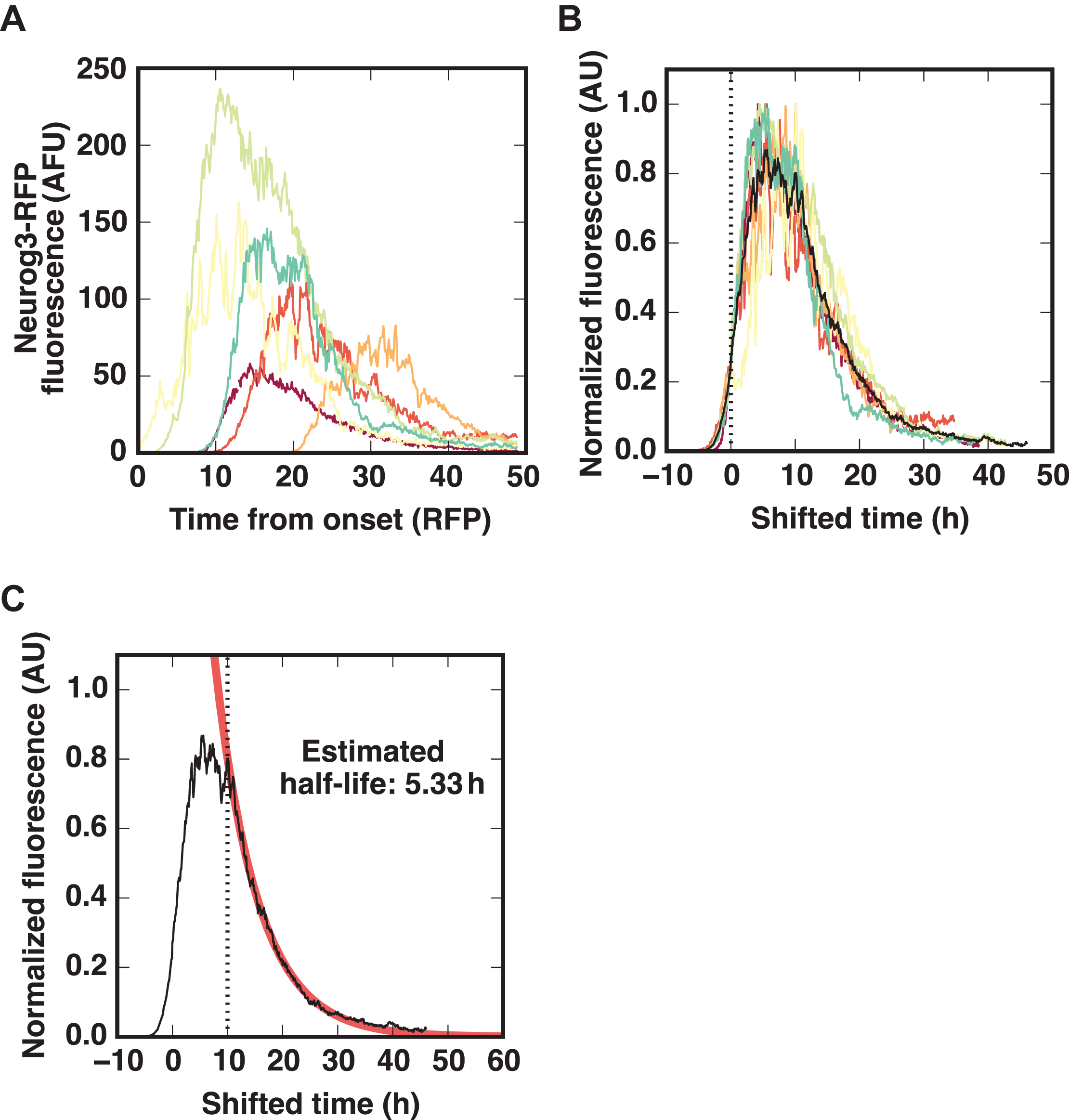

Supplement: S5 Fig — (A) Fluorescence intensity of individual Neurog3-RFP cells over time. Different coloured lines indicate individual RFP cells. (B) Normalized fluorescence of all the RFP signals from (A) and aligned by 25% intensity to time 0. Black line indicates average intensity of all RFP signals. (C) Estimation of RFP half-life. Red line indicates the trend of exponential decay. RFP half-life is estimated as 5.3 h. (TIF) [file pbio.1002111.s007.tif]

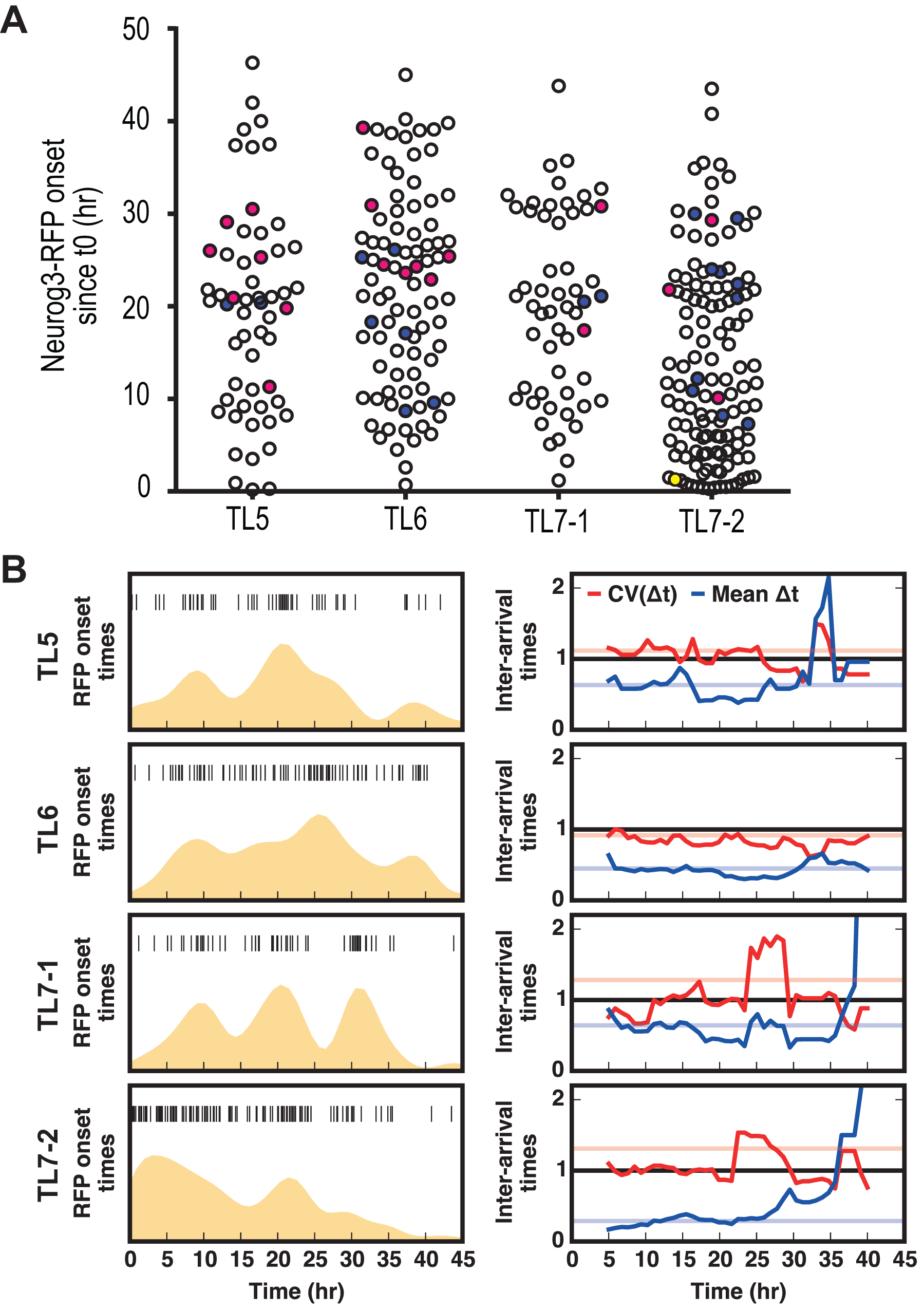

Supplement: S6 Fig — (A) RFP onset time distribution from four time-lapse movie positions (n = 56, 89, 54, and 125). Each circle indicates onset time of RFP cell. Red, blue, and yellow circles indicate RFP cells arising from asymmetric, symmetric, and RFP divisions, respectively. (B) Analyses of RFP onset Coefficient of Variance and sliding window for oscillatory patterns. The Coefficient of Variance of each time-lapse onset distribution is equivalent to a homogeneous process, which is equal to 1. (TIF) [file pbio.1002111.s008.tif]

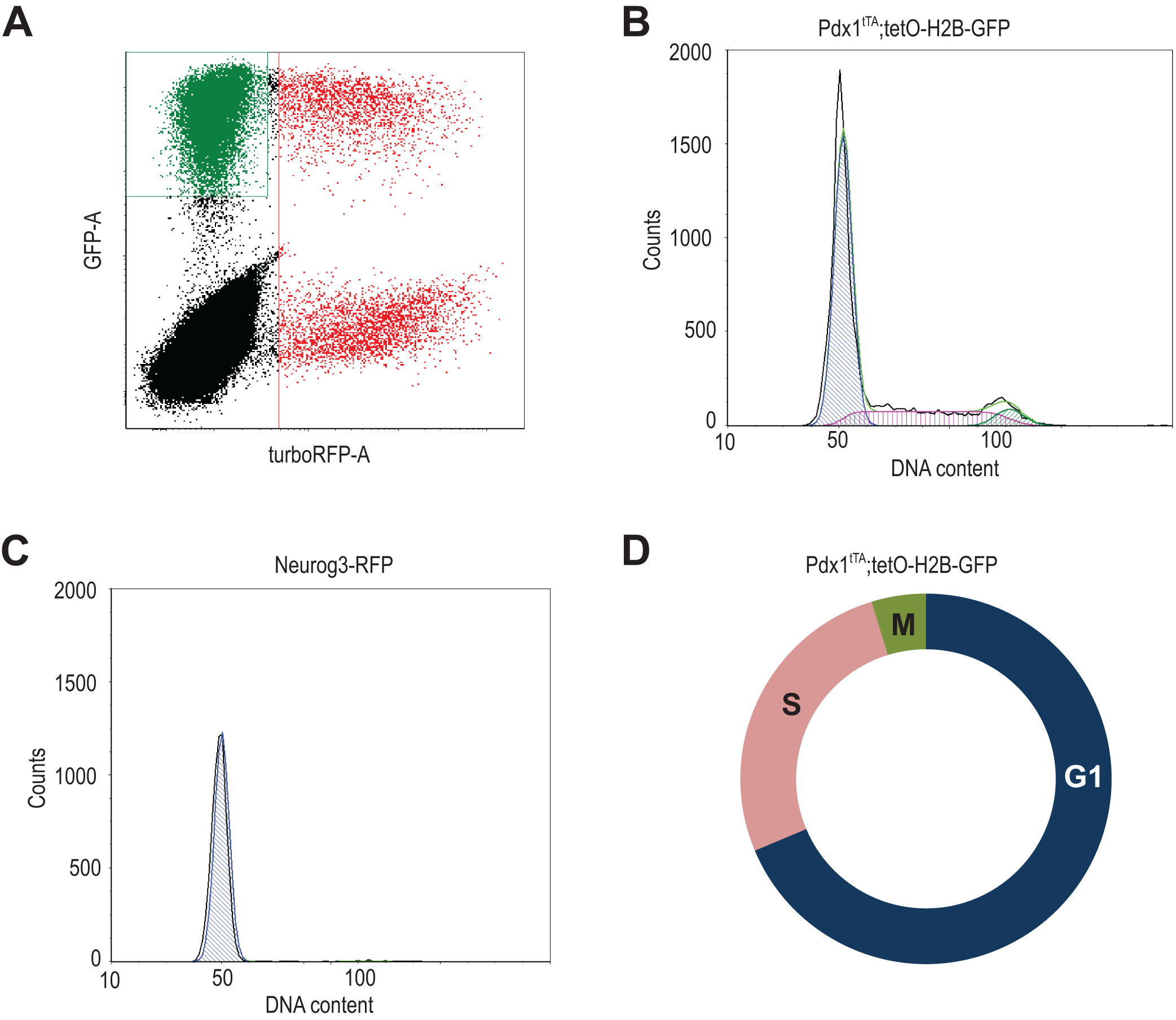

Supplement: S7 Fig — (A) Flow cytometry of dissociated pancreatic cells from pooled pancreata from a litter (10 embryos: 2 GFP+/RFP+, 1 GFP+, 1 RFP+, and 6 negative pancreata) for DNA content by DAPI staining. (B) Cell cycle analysis of pancreatic progenitors (Pdx1tTA/+;tetO-H2B-GFP) by DAPI-stained DNA content. This panel shows 66.3% of cells in G1/G0 phase, 29.2% in S phase, and 4.5% in G2/M phase. (C) Cell cycle analysis of endocrine progenitors (Neurog3-RFP) by DAPI-stained DNA content. Note 97.2% of cells in G1/G0 phase, 1.6% in S phase and 1.2 in G2/M phase, as endocrine progenitors are mostly post-mitotic. (D) Average cell cycle of pancreatic progenitors (n = 3). 68.7% ± 2.1% of cells are in G0/G1, 26.6% ± 2.4% in S phase, and 4.7% ± 1.2% in G2/M phase. (TIF) [file pbio.1002111.s009.tif]

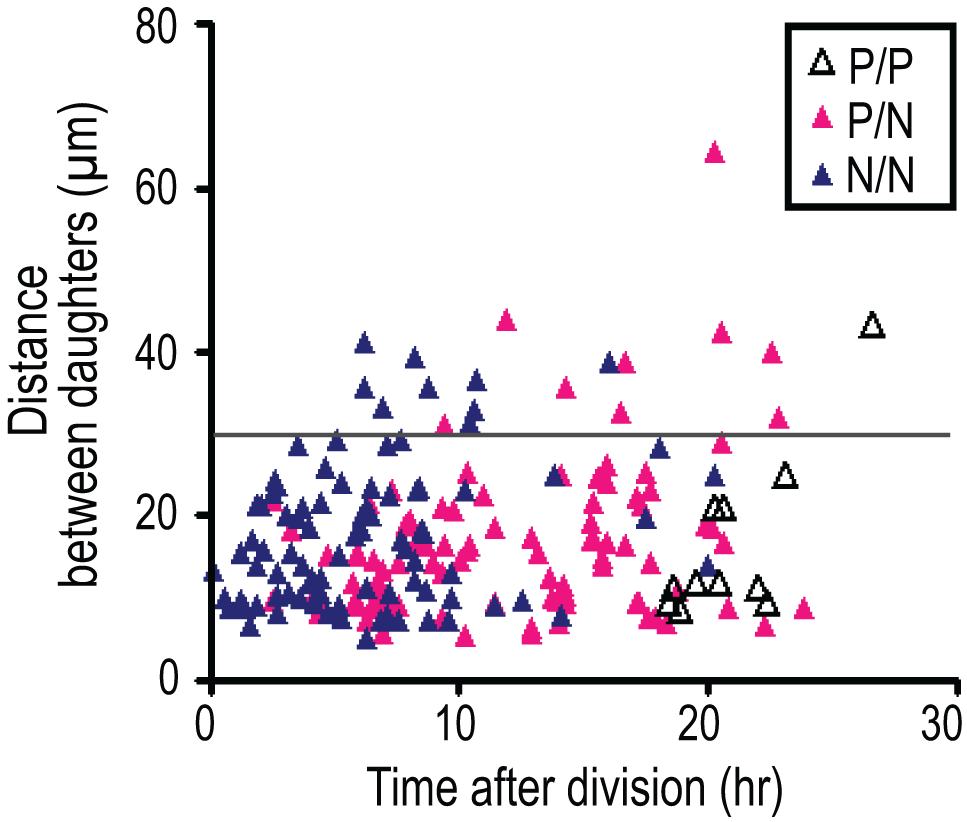

Supplement: S8 Fig — The x-axis represents the time between division and NEUROG3 end-point immunostaining. White triangles show the distance between two progenitor daughters originating from symmetric divisions (P/P), pink triangles, distance between one progenitor and one NEUROG3 cell from asymmetric divisions (P/N), and blue triangles, distance between two NEUROG3 cells from symmetric divisions (N/N). Distance from P/P divisions was measured one frame prior to daughter divisions, which indicates doubling time between mother and daughter divisions. There is therefore no data point before 18 h. Regardless of the cell division mode, the distance between daughters increased, as time after division increased (Pearson’s r, 0.81 [P/P], 0.33 [P/N], and 0.27 [N/N]). All three different modes of division showed statistically significant correlations between daughter distance and time: P/P division, p = 0.0025, P/N division, p = 0.0012, and N/N division, p = 0.016. The grey line indicates 30 μm threshold used for in vivo clonal analysis for two-cell clone boundaries. 90.2% of all data are under this threshold. (TIF) [file pbio.1002111.s010.tif]

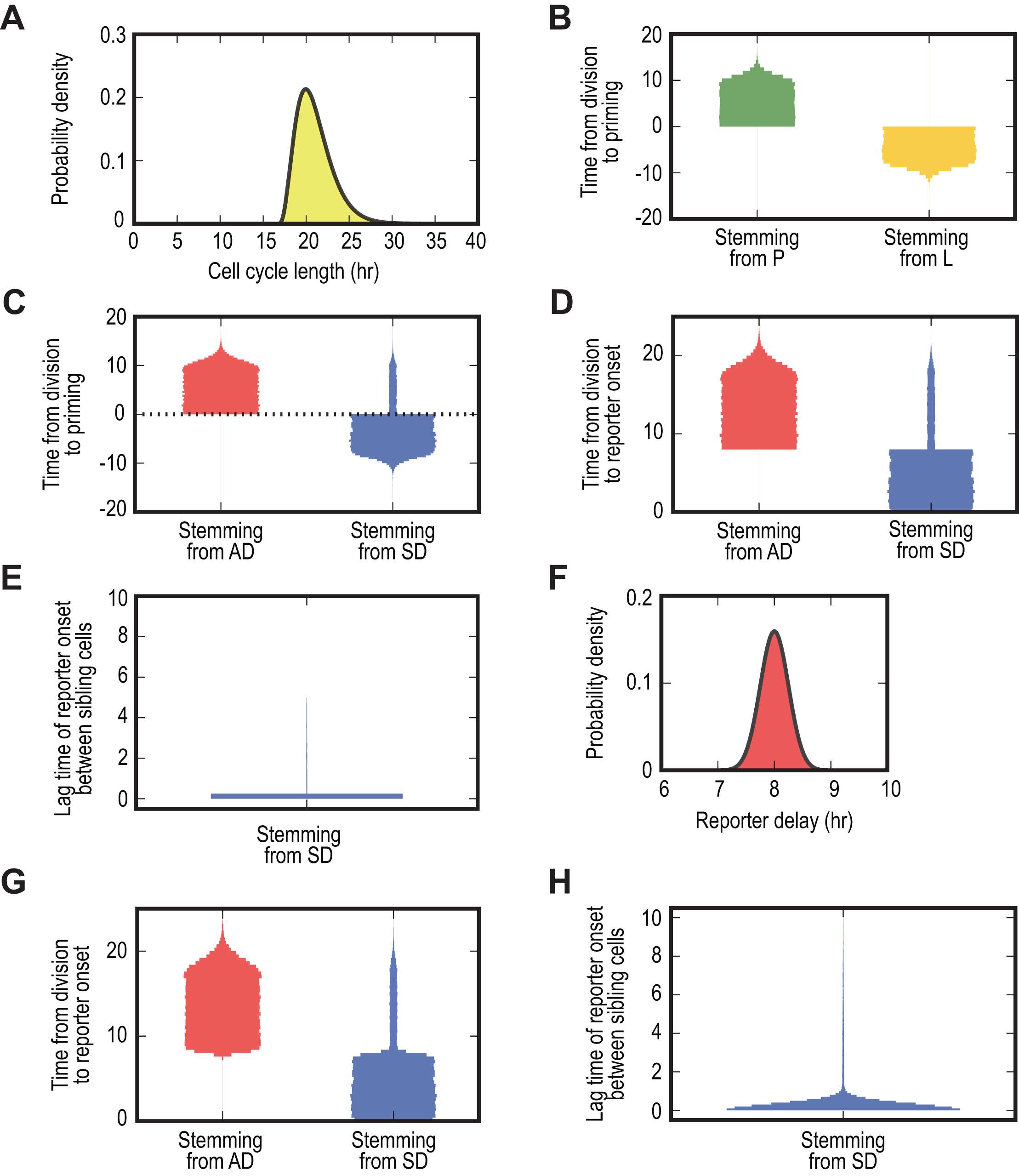

Supplement: S9 Fig — Monte Carlo simulations of clone expansion according to the model. Each newly born cell is randomly assigned a cell cycle length from a Gamma shifted (A) with mean and variance matching the experimentally measured. (B) Distribution of times from division to the priming event for cells stemming from P and L cells. Note that in the latter the priming event occurs prior to division. (C) Distribution of times from division to the priming event for cells stemming from AD and SD divisions. (D) Distribution of set times for a reporter with constant delay. (E) Lag time between the reporter onset in sibling cells. The simulations anticipate a high degree of synchronization. (F–H) Effect of heterogeneity in the reporter delay. (F) The distribution of delays of the reporter onset is assumed Gaussian. Variability in the reporter delay has little impact in the distribution of lag times in ACD and SCD originated cells (G) and in the synchronization of sibling cells (H). (TIF) [file pbio.1002111.s011.tif]

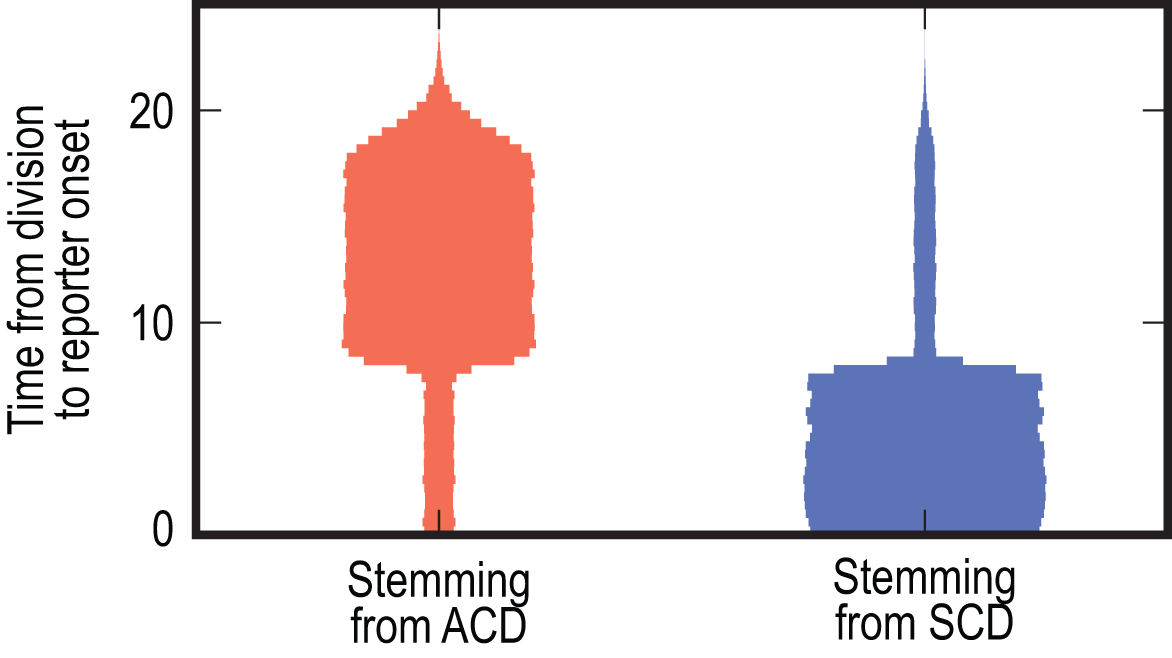

Supplement: S10 Fig — In order to account for the effect of possible misclassification biases in the distribution of lag times, we performed an in silico misclassification experiment. We randomly sampled 11.6% of simulated cells stemming from symmetric divisionand classified them as stemming from asymmetric division. The resulting distribution of reporter onset lag times for this latter group features a tail at low times that might partly account for the observed (cf. Fig. 4C). This tail in the distribution, however, does not substantially affect the average lag time. (TIF) [file pbio.1002111.s012.tif]

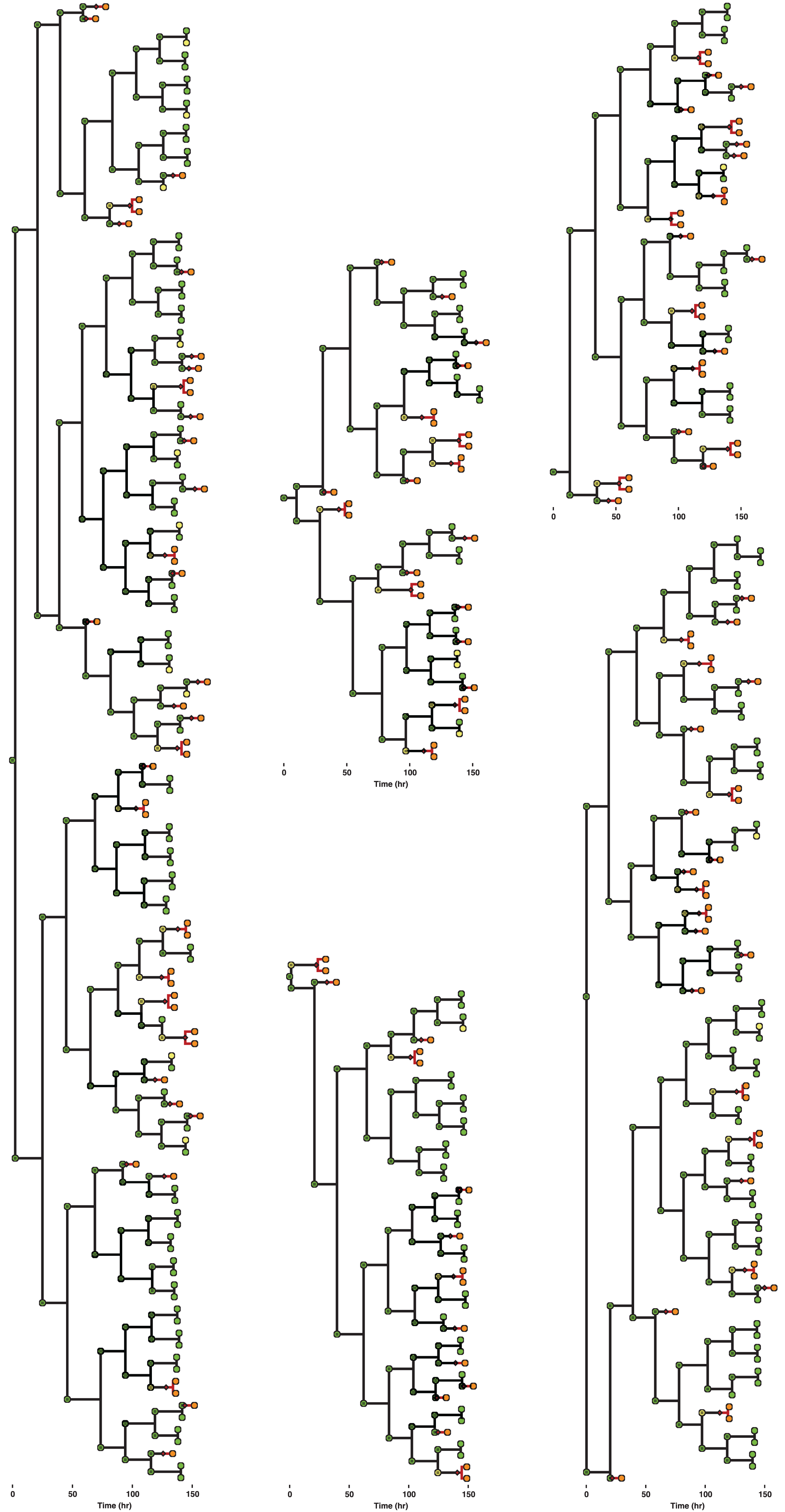

Supplement: S11 Fig — Temporal evolution of the expansion of five representative clones obtained with Monte-Carlo simulations of the model (q = 0.2, θ = 0.55). X- axis indicates experimental time. All simulations start at time zero with one progenitor cell (P, green circle) whose cell cycle phase is set uniformly at random and run for 150 h. Progenitor cells continuously divide according to the distribution of cell cycles in S9A Fig.. Stochastic priming events are indicated by red diamonds. Events occurring early following cell division lead to cells exiting the cell cycle and differentiating (N cells, orange circles). Events taking place later on lead to primed cells that are already committed to complete the cell cycle (L cells, yellow circles) and that will give rise to two differentiated post-mitotic cells (N cells). (TIF) [file pbio.1002111.s013.tif]
