# Supplementary material for: Cell Cycle–Dependent Differentiation Dynamics Balances Growth and Endocrine Differentiation in the Pancreas
Source: PLoS Biol. 2015 Mar 18;13(3):e1002111. doi: 10.1371/journal.pbio.1002111 (PMC4364879; doi:10.1371/journal.pbio.1002111)
Supplement: S1 Table — (DOCX) [file pbio.1002111.s022.docx]

**S1 Table. Fraction of NEUROG3 over Pdx1^tTA/+^;tetO-H2B-GFP cells from immunostaining images after time-lapse imaging.**

|  | TL1 | TL2 | TL3 | TL4 | Mean | SD | Control* | E14.5 WT** |
| --- | --- | --- | --- | --- | --- | --- | --- | --- |
| Ngn3 # | 228 | 333 | 298 | 243 |  |  | 154 |  |
| H2B-GFP # | 7069 | 7090 | 5608 | 6644 |  |  | 6145 |  |
| Ngn3/GFP | 0.032 | 0.047 | 0.053 | 0.037 | 0.042 | 0.0095 | 0.025 | 0.089±0.009 |

*Explant control without imaging.

**Ngn3/PDX1 measurements (Mean ± standard deviation) from E14.5 pancreata (n=4).
