# Supplementary material for: Cell Cycle–Dependent Differentiation Dynamics Balances Growth and Endocrine Differentiation in the Pancreas
Source: PLoS Biol. 2015 Mar 18;13(3):e1002111. doi: 10.1371/journal.pbio.1002111 (PMC4364879; doi:10.1371/journal.pbio.1002111)
Supplement: S2 Table — (DOCX) [file pbio.1002111.s023.docx]

**S2 Table. Data from NEUROG3^+^ cell tracking in time-lapse movies (Pdx1^tTA/+^*;tetO-H2B-GFP*).**

|  | TL1 |  | TL2 |  | TL3 |  | TL4 |  | Total |  |
| --- | --- | --- | --- | --- | --- | --- | --- | --- | --- | --- |
| Imaging duration (hr:mm) | 18:36 |  | 18:48 |  | 24:00 |  | 24:06 |  |  |  |
| total div* | 221 |  | 546 |  | 250 |  | 611 |  | 1628 |  |
| Ngn3-prod. div* | 19 |  | 38 |  | 15 |  | 30 |  | 102 |  |
| Back-traced | Division | Ngn3 # | Division | Ngn3 # | Division | Ngn3 # | Division | Ngn3 # | Division | Ngn3 # |
| Asym div (P/N) | 24 | 24 | 24 | 24 | 21 | 21 | 36 | 36 | 105 | 105 |
| Sym div (N/N) | 17 | 34 | 30 | 60 | 7 | 14 | 40 | 80 | 94 | 188 |
| sister lost | 11 | 11 | 1 | 1 | 7 | 7 | 5 | 5 | 24 | 24 |
| sister dead | 9 | 9 | 4 | 4 | 6 | 6 | 6 | 6 | 25 | 25 |
| No div |  | 55 |  | 52 |  | 28 |  | 36 |  | 171 |
| lost |  | 42 |  | 18 |  | 10 |  | 12 |  | 82 |
|  |  |  |  |  |  |  |  |  |  |  |
| Total | 61 | 175 | 59 | 159 | 41 | 86 | 87 | 175 | 248 | 595 |

* Number of division counted in a cropped position.
