# Supplementary material for: Cell Cycle–Dependent Differentiation Dynamics Balances Growth and Endocrine Differentiation in the Pancreas
Source: PLoS Biol. 2015 Mar 18;13(3):e1002111. doi: 10.1371/journal.pbio.1002111 (PMC4364879; doi:10.1371/journal.pbio.1002111)
Supplement: S3 Table — (DOCX) [file pbio.1002111.s024.docx]

**S3 Table. Data from *in vivo* clonal analysis (*Hnf1bCreER;mT/mG*).**

| Sample ID | 1 | 2 | 3 | 4 | 5 | 6 | 7 | 8 | 9 | 10 | 11 | 12 | 13 | 14 | 15 | 16 | 17 | 18 | 19 | 20 | 21 | 22 | total |
| --- | --- | --- | --- | --- | --- | --- | --- | --- | --- | --- | --- | --- | --- | --- | --- | --- | --- | --- | --- | --- | --- | --- | --- |
| No. Of Recombined cells* | 18 | 65 | 18 | 61 | 45 | 95 | 60 | 40 | 57 | 40 | 39 | 60 | 38 | 65 | 48 | 61 | 19 | 31 | 118 | 63 | 108 | 16 | 1165 |
| No. of 2-cell clones | 4 | 18 | 5 | 16 | 10 | 20 | 12 | 10 | 14 | 10 | 10 | 11 | 8 | 13 | 10 | 15 | 2 | 4 | 18 | 12 | 20 | 2 | 244 |
| SOX9^+^/SOX9^+^ | 1 | 13 | 3 | 8 | 6 | 14 | 7 | 5 | 6 | 5 | 5 | 6 | 4 | 8 | 6 | 9 | 1 | 4 | 10 | 10 | 14 | 1 | 146 |
| SOX9^+^/NEUROG3^+^ | 0 | 0 | 0 | 1 | 4 | 0 | 3 | 2 | 4 | 3 | 2 | 1 | 1 | 2 | 1 | 5 | 0 | 0 | 1 | 1 | 3 | 0 | 34 |
| NEUROG3^+^/NEUROG3^+^ | 0 | 2 | 0 | 2 | 0 | 1 | 0 | 1 | 2 | 0 | 1 | 1 | 1 | 1 | 2 | 1 | 0 | 0 | 6 | 0 | 0 | 0 | 21 |
| NEUROG3^+^/NEUROG3^-^SOX9^-^ | 0 | 0 | 0 | 1 | 0 | 2 | 0 | 1 | 2 | 1 | 0 | 0 | 0 | 1 | 0 | 0 | 0 | 0 | 0 | 0 | 0 | 0 | 8 |
| SOX9^+^/SOX9^-^NEUROG3^-^ | 2 | 1 | 0 | 3 | 0 | 0 | 1 | 0 | 0 | 1 | 1 | 2 | 2 | 1 | 1 | 0 | 0 | 0 | 1 | 0 | 1 | 0 | 17 |
| SOX9^-^NEUROG3^-^/ SOX9^-^NEUROG3^-^ | 1 | 2 | 2 | 1 | 0 | 3 | 1 | 1 | 0 | 0 | 1 | 1 | 0 | 0 | 0 | 0 | 1 | 0 | 0 | 1 | 2 | 1 | 18 |

*mostly 1-cell clones except 14 3-cell clones and 2 4-cell clones out of 1165 recombined cells.
