# Supplementary material for: Cell Cycle–Dependent Differentiation Dynamics Balances Growth and Endocrine Differentiation in the Pancreas
Source: PLoS Biol. 2015 Mar 18;13(3):e1002111. doi: 10.1371/journal.pbio.1002111 (PMC4364879; doi:10.1371/journal.pbio.1002111)
Supplement: S4 Table — (DOCX) [file pbio.1002111.s025.docx]

**S4 Table. Data from *in vitro* clonal analysis (*Hnf1bCreER;mT/mG*).**

| Sample ID | 1 | 2 | 3 | 4 | 5 | 6 | 7 | 8 | 9 | 10 | 11 | Total | Frequency |
| --- | --- | --- | --- | --- | --- | --- | --- | --- | --- | --- | --- | --- | --- |
| 2-cell clones | 11 | 13 | 0 | 9 | 10 | 7 | 9 | 9 | 9 | 8 | 11 | 96 | - |
| SOX9 SCD | 11 | 10 | 0 | 6 | 7 | 6 | 7 | 8 | 9 | 8 | 7 | 79 | 0.8229167 |
| NEUROG3/SOX9 ACD | 0 | 2 | 0 | 0 | 0 | 0 | 1 | 1 | 0 | 0 | 1 | 5 | 0.0520833 |
| NEUROG3 SCD | 0 | 0 | 0 | 0 | 0 | 0 | 0 | 0 | 0 | 0 | 1 | 1 | 0.0104167 |
| SOX9+/SOX9- | 0 | 1 | 0 | 2 | 2 | 1 | 1 | 0 | 0 | 0 | 1 | 8 | 0.0833333 |
| SOX9-/SOX9- | 0 | 0 | 0 | 1 | 1 | 0 | 0 | 0 | 0 | 0 | 1 | 3 | 0.03125 |
