# Supplementary material for: Cell Cycle–Dependent Differentiation Dynamics Balances Growth and Endocrine Differentiation in the Pancreas
Source: PLoS Biol. 2015 Mar 18;13(3):e1002111. doi: 10.1371/journal.pbio.1002111 (PMC4364879; doi:10.1371/journal.pbio.1002111)
Supplement: S5 Table — (DOCX) [file pbio.1002111.s026.docx]

**S5 Table. Data from Neurog3-EYFP;Neurog3-RFP explant time-lapse.**

|  | exp1 | exp2 | exp3 | Total | avg | stdev |
| --- | --- | --- | --- | --- | --- | --- |
| YFP+ # @t0 | 178 | 134 | 165 | 477 |  |  |
| RFP+ # @t0 | 153 | 110 | 141 | 404 |  |  |
| RFP+ # after t0 | 6 | 3 | 10 | 19 |  |  |
| potent. double (+)*# | 159 | 113 | 151 | 423 |  |  |
| YFP+RFP- # @t0 | 19 | 21 | 14 | 54 |  |  |
| RFP+/YFP+ ratio @t0 | 0.86 | 0.82 | 0.85 |  | 0.84 | 0.02 |
| YFP+/RFP+ ratio @t0 | 1 | 1 | 1 |  | 1 |  |
| double (+)*/YFP+ ratio | 0.89 | 0.84 | 0.92 |  | 0.88 | 0.04 |
| RFP-/YFP+ ratio | 0.11 | 0.16 | 0.08 |  | 0.12 | 0.04 |

*Potentially double positive cells that acquire RFP expression at later time points from time-lapse movies, although the cells were initially YFP^+^/RFP^-^ at time 0.
