# Supplementary material for: Cell Cycle–Dependent Differentiation Dynamics Balances Growth and Endocrine Differentiation in the Pancreas
Source: PLoS Biol. 2015 Mar 18;13(3):e1002111. doi: 10.1371/journal.pbio.1002111 (PMC4364879; doi:10.1371/journal.pbio.1002111)
Supplement: S6 Table — (DOCX) [file pbio.1002111.s027.docx]

**S6 Table. Data from RFP^+^ cell tracking in time-lapse movies (Pdx1^tTA/+^*;tetO-H2B-GFP;Neurog3-RFP*).**

|  | TL5 |  | TL6 |  | TL7 |  | Total |  |
| --- | --- | --- | --- | --- | --- | --- | --- | --- |
| Imaging duration (hr:mm) | 48:30 |  | 47:54 |  | 48:42 |  |  |  |
| Back-traced | Division | RFP # | Division | RFP # | Division | RFP # | Division | RFP # |
| Asym div (P/N) | 11 | 11 | 9 | 9 | 7 | 7 | 27 | 27 |
| Sym div (N/N) | 6 | 12 | 4 | 8 | 8 | 16 | 18 | 36 |
| sister lost* | 3 | 3 | 5 | 5 | 3 | 3 | 11 | 11 |
| sister dead | 3 | 3 | 1 | 1 | 2 | 2 | 6 | 6 |
| No division |  | 49 |  | 17 |  | 20 |  | 86 |
| Lost** |  | 146 |  | 160 |  | 103 |  | 409 |
| RFP+ cell division | 2 | 4 | 0 | 0 | 1 | 2 | 3 | 6 |
| Total | 25 | 228 | 19 | 200 | 21 | 153 | 65 | 581 |

*Mostly due to low GFP signal.

**Mostly due to moving out of Z position during backtracking. i.e. During the acquisition of images, the explants flatten, therefore, the majority of lost RFP cells appear during the flattening process over time.
