# Supplementary material for: Cell Cycle–Dependent Differentiation Dynamics Balances Growth and Endocrine Differentiation in the Pancreas
Source: PLoS Biol. 2015 Mar 18;13(3):e1002111. doi: 10.1371/journal.pbio.1002111 (PMC4364879; doi:10.1371/journal.pbio.1002111)
Supplement: S7 Table — (DOCX) [file pbio.1002111.s028.docx]

**S7 Table. Primary antibodies used for immunostainings.**

| Epitope | Species generated | Dilution factor | Provider | Catalog number |
| --- | --- | --- | --- | --- |
| NEUROG3 | goat | 1:1,000 | BCBC* | AB2774 |
| SOX9 | rabbit | 1:2,000 | Millipore | AB5535 |
| aPKC | rabbit | 1:1,000 | Santa Cruz | sc-216 |
| E-CAD | mouse | 1:200 | BD Trasduction Lab | C20820 |
| MYC | mouse | 1:200 | Invitrogen | 13-2500 |
| GFP | chicken | 1:1,000 | Abcam | AB13970 |
| INSULIN | guinea pig | 1:50 | Dako | A0564 |
| GLUCAGON | guinea pig | 1:800 | Linco | 4031-01F |

*BCBC, Beta Cell Biology Consortium, funded by NIDDK U01DK072473.
