# Supplementary material for: Cell Cycle–Dependent Differentiation Dynamics Balances Growth and Endocrine Differentiation in the Pancreas
Source: PLoS Biol. 2015 Mar 18;13(3):e1002111. doi: 10.1371/journal.pbio.1002111 (PMC4364879; doi:10.1371/journal.pbio.1002111)
Supplement: S1 Text — (DOCX) [file pbio.1002111.s029.docx]

**Supporting Information**

**S1 Text. Supplemental methods**

**S1.1 Text. Cell cycle analysis**

Pooled pancreata from 1 or 2 litters of E14.5 embryos with different genotypes from Pdx1^tTA/+^*;Neurog3-RFP x* *tetO-H2B-GFP* breeding were dissociated mechanically using 0.05% Trypsin containing DNase I (200 U/ml), and fixed in 4% PFA. After washing with PBS, the dissociated cells were nuclear-stained by DAPI (1 μg/ml). Then cell cycle analysis was performed by sorting GFP^+^ and RFP^+^ single cells by LSRFortessa cell analyzer (BD Bioscences). Cell cycle curve fitting and calculation were done using FCS express software (De Novo Software).

**S1.2 Text. Time-lapse imaging with Neurog3-EYFP;Neurog3-RFP pancreata**

Pancreatic explant culture and live imaging were performed as described in the Materials and Methods, with imaging modifications of 10 minute intervals for up to 65 hours using a 20X glycerol-immersion objective.

**S1.3 Text. Quantitative characterization of transient Neurog3-RFP response in differentiating cells**

To study the dynamics of cells during fate specification we performed live tracking of single cells using a novel Neurog3-RFP reporter. As a first step, we quantitatively characterized the response of the fluorescent reporter upon cell differentiation. We extracted fluorescence time traces of individual cells at a high temporal resolution (10 frames/hour) and used the average signal intensity over a spherical region of interest of radius (5 µm) approximately located around the center of cell nuclei. We selected those cells for which we could completely track the response of the signal. S5A Fig. shows the RFP fluorescence traces for those cells (n=6). All cells displayed a transient rise in fluorescence followed by a rapid descent. Despite the maximum signal amplitude varied from cell to cell, the shape of the transient response was fairly conserved amongst cells. To confirm this pattern we aligned signals in time and normalized their maximum amplitude to one. With this transformation fluorescence signals from all cells collapsed into one stereotypical response curve (S5B Fig.), a pulse consisting a rapid increase in fluorescence followed by an exponential decay. According to this shape, the total persistence of the fluorescence after the onset is of about 20-30 hours. Fitting an exponential to the decay we estimated an effective half-life of 5-6 hours for the RFP (S5C Fig.). Such fast turnover indicates that the perdurance of the RFP signal might be due to constant transcription.

Another important aspect of the dynamics of the RFP signal in reporting the cell differentiation state is the delay between the priming event and the reporter onset. Analysis of cells bearing both EYFP and RFP reporters indicates that the latter is delayed by about 2.8 to 6.3 hours with respect to the former (S4D,E Fig.). Because the onset of the EYFP reporter might also be delayed with respect to the priming event, we consider the estimated range to be a lower boundary of the true delay between the differentiation priming and the onset of the RFP reporter.

**S1.4 Text. Analysis of tissue level coordination of cell differentiation**

Live imaging of the RFP reported onset allowed us to detect individual cells undergoing NEUROG3 differentiation within the expanding tissue. An initial analysis of the RFP onset events pointed towards discrete waves of differentiation. This is best illustrated by the undulated profiles of the cell differentiation probability. These profiles were obtained by gaussian kernel density estimation (bandwidth: 10 minutes) and depicted in Fig. 4A (reproduced in S6B Fig.). However, because the results of kernel density estimation depend on the selected bandwidth, we decided to further analyze the statistics of the time intervals between consecutive differentiating cells. To do so, we compared our data to a homogeneous Poisson process. In such process each event (i.e., the RFP onset of one cell) is independent of the others, the rate of events is constant over time and the time between consecutive events has an exponential distribution. This means that the average time between consecutive events is equal to its standard deviation and hence the coefficient of variation (standard deviation normalized to the mean, CV) is equal to one. Using the data of four explant locations during the initial 35 hours of time-lapse movies (right panels in S6B Fig.), we estimated the CV of the time between consecutive RFP onset events to be close to one (1.16±0.08). We also computed the CVs using a sliding window of width 10 hours and found that this is maintained around one most of the time (S6B Fig., left panels). Hence, with these results we cannot completely rule out the possibility that the observed patterns of differentiation are mere instances of a homogeneous process.

**S1.5 Text. Estimation of the probability of differentiation and association between cell cycle and differentiation priming**

In tissues with only two cellular types, namely a proliferating and a differentiated fate, each cell division can be classified into either symmetric proliferating (SP) if the two newly formed cells are proliferative (*P* cells); symmetric differentiating (SD) if the two daughters differentiate (*N* cells), or asymmetric (AD) divisions if there is one daughter of each kind.

This classification provides a straightforward way to indirectly estimate the probability of cell differentiation, *q*, given the probabilities (frequencies) of division types:

*q =* Pr[*SD*] *+ ½* Pr[*AD*]*.*

That is to say, the probability of differentiation can be regarded as the contribution of all cells stemming from SD divisions plus half of the cells from asymmetric divisions.

It is important to note, however, that the relation between the probability of differentiation *q* and the frequency of each division type is not unambiguous, i.e., there are infinitely many combinations of Pr[*SP*], Pr[*SD*] and Pr[*AD*] that lead to the same probability of differentiation *q*. Thus, given only *q* we cannot infer the frequencies of each division type.

However, with the proportions of each division type we can test whether the fate adopted by pairs of sibling cells is statistically independent or tied. In the case where sibling cells decide to differentiate independently of each other, the frequencies of the cell division modes follow a binomial relation as in the case of allele frequencies of a population in Hardy-Weinberg equilibrium:

Pr[*SD*] *= q^2^*, Pr[*AD*] *= 2q(1-q),* and Pr[*SP*] *= (1-q)^2^*

Lack of independence (or linkage disequilibrium) can be statistically assessed by means of contingency table tests such as Pearson’s χ^2^ or Fisher’s exact test.

In the case developing pancreatic progenitors, we estimated the proportions of SP (P/P), AD (P/N) and SD (N/N) division from the *in vivo* clonal analysis (Fig. 2H). These are 69.9%, 18.6% and 11.5%, respectively. From these we estimate the probability of cell differentiation to be *q*=0.208. That is to say, around 20.8% of the newly born cells will eventually differentiate. This probability of differentiation implies a net exponential growth of the tissue (q<0.5). If these differentiation events were independent in sibling cells, the proportions of each division type would be 62.7% for *SP divisions*, 33% for AD and only 4.3% for SD divisions. These proportions are far from the observed ones thus suggesting that fates of sibling cells are statistically linked. This is further confirmed with statistical tests on the absolute numbers of divisions recorded (*p<10^-7^* for both Pearson’s χ^2^ or Fisher’s exact tests). Note that even though the above proportions have not been directly computed from the recorded raw numbers of divisions of each type (SP: 146, SD: 34, SD: 21), the raw relative amounts of each division mode do not differ from the above estimates (146/201=72.6% vs 69.9%, 33/201=17% vs 18.6% and 21/201=10.4% vs 11.5%) and nor does *q (3.6%* vs *4.3%).*

**S1.6 Text. Mathematical modeling**

In order to gain conceptual and quantitative insight into the cell proliferation and differentiation dynamics during pancreas development, we conceived a simple model of cell cycle-dependent stochastic priming of progenitors to endocrine fate. The model considers three different cell types: pancreatic progenitors (P), post-mitotic Neurog3 cells (N), and cells primed for differentiation that will complete the division cycle (L cells). Each newly formed cell from a dividing P cell will adopt one of the three fates above with certain probabilities: a cell might differentiate with probability *q* or remain as a P cell with probability *1 - q*. Furthermore, a differentiating cell might either adopt N or L fate with probabilities *θ* and *1- θ*, respectively (Fig. 5D). All L cells eventually divide generating two N cells. Note that the cellular state L is transient and thus from an experimental point of view L cells might not be distinguishable from P cells. These rules lead to seven different division events that can be classified into the three modes generally considered:

- **SP divisions:** *P → P + P*, *P → L + L* and *P → P + L*,
- **AD divisions:** *P → L + N* and *P → P + N*, and
- **SD divisions:** *P → N + N* and *L → N +N*.

We have formally stated and analyzed these events in terms of a model of discrete-time dynamic equations - mathematical details can be found elsewhere[[33](#_ENREF_33)]. Here we briefly summarize the model results.

- Given the parameters *q* and *θ,* the frequencies of each division mode are:
  Pr[*SP*] *= (1-q)(1-qθ)*, Pr[*SD*] *= q(1-θ(1-q))*, and Pr[*AD*] *= (1-q)2qθ.*
- Conversely, the parameters *q* and *θ* can be inferred from the experimentally observed frequencies of each division mode: *q =* Pr[*SD*] *+ ½* Pr[*AD*] and *θ = ½* Pr[*AD*]/(q(1-q)). Therefore, from the *in vivo* data we estimate *q=0.208* and *θ=0.565.*
- The long-term composition of the tissue can also be predicted from the model. The fractions of each cell type are given by the following equations *π_P_=(1-2q)(1-q)/(1- qθ)*, *π_L_=(1-2q)(q-qθ)/(1- qθ)* and *π_N_=2q.* For the experimentally estimated values the model predicts that 52.5% of the cells are progenitors, 41.5% are post-mitotic cells, and only 6% of the cells are of the type L (i.e., they are committed to differentiation but will divide once).
- The model also discriminates between those SD divisions that are due to an L cell from those that occur in P cells when the two daughter cells independently adopt the N fate. The fraction of divisions of the former type is given by:
   Pr[mother cell is *L | SD*] = *(qθ-q^2^θ^2^)/(1 – θ + qθ - qθ^2^)*, and
   Pr[mother cell is *P | SD*] = *(1 - θ)/(1 – θ + qθ - qθ^2^).*With our data we estimate these proportions to be 90.2% vs 9.8%. That is to say, the vast majority of SD divisions are due to cells that are already primed for differentiation before division.

In addition to these predictions, the model can also prognosticate the dynamics of cell differentiation. For that to occur we need to interpret the model in a specific manner by linking the probability *θ* to the cell cycle phase. In particular, we need to assume that progenitor cells are susceptible to be primed for differentiation within a window of the cell cycle (which could correspond to the whole cycle or to a specific phase). In addition, this priming event might have different outcomes depending on whether it occurs at the beginning or the end of this time window. If this time window has a length of one, then *θ* might represent the time when the cell commits to cell cycle completion. Any priming event before *θ* implies the exit from cell cycle and terminal differentiation (N state adoption), and any event after *θ* involves a cell that will divide into two differentiated cells (L state adoption). Therefore, if the probability of being primed is approximately uniform throughout the time window, then the model presented above precisely describes this system. This interpretation in terms of priming time implies further testable predictions. Hereafter we assume *<T>* to be the average cell cycle and the susceptibility window to span the whole cell cycle duration:

- Obviously, sibling cells stemming from L cells will be perfectly synchronized, as there is only one priming event common to both cells (it occurs in the mother L cell). Hence, according to the model, a perfect synchronization is expected in at least 93.8% of all SD divisions (see above).
- On average, the time between birth and priming in differentiated cells stemming from a P cell is *<Δt_P_>=½θ<T>.* On the other hand, in differentiated cells stemming from a L cell this time becomes *<Δt_L_>=-½(1-θ)<T>* (i.e., the priming event occurs before division). Therefore, on average, N cells stemming from L cells will anticipate their differentiation programme by about *<Δt_P_>-<Δt_L_>=½<T>* (i.e., half a cell cycle) the cells from stemming from P cells.
- On average, lag time between birth and priming in differentiated cells stemming from asymmetric divisions is *<Δt_AD_>=<Δt_P_>* (all cells stem from a P cell). In cells stemming from SD divisions, this time becomes *<Δt_SD_>=<Δt_P_>* Pr[mother cell is *P | SD*]  *+ <Δt_L_>* Pr[mother cell is *L | SD*]≈*<Δt_L_>,* and therefore cells from SD divisions anticipate those from AD divisions in differentiation by approximately *½<T>*.

Despite the simplicity of the model, the generated predictions are approximately fulfilled by the experimental data (see Fig. 5), thus suggesting that the model might be capturing the dynamics of cell fate adoption during pancreas development.

We decided to include more details in the model in order to generate more quantitatively accurate predictions and compare them to the experimental data. We proceeded by performing Monte Carlo simulations of the model including the observed variability in the cell cycle length and the delay in the onset of the reporter. In the simulations each newly born cell *n* is randomly assigned a cell cycle length *T_n_* from a Gamma shifted distribution (shift 17, shape 4, scale 1). This distribution (S9A Fig.) has a mean time of 21 hours and standard deviation of 2 hours, which closely resemble those measured experimentally (Fig. 4D). These cells become randomly primed with probability *q*. Once a cell is primed for differentiation, it is assigned a delay *δ_rep_* for the onset of the reporter. In the first set of simulations we consider this delay to be fixed to 8 hours, which we consider to be a reasonable delay for the RFP (see previous section for an estimate of the lower boundary of this delay). For each cell primed for differentiation, a random number *r* between 0 and 1 is drawn, and the priming event is considered to occur at time *T_n_r* after cell birth. If *r<θ* then the cell differentiates into an N cell (i.e., becomes post mitotic). Conversely, if *r≥θ* the cell commits to cell cycle completion and divides at time *T_n_*, giving rise to two N cells. It can be shown that the average behaviour of this computational model corresponds to the analytic model described above.

Individual clones starting from an individual progenitor cell are simulated for a period of 150 hours. S11 Fig. shows a graphical representation of 5 simulated clones. The results from the simulation of 10,000 clones are displayed in S9 Fig. Panel B in this figure shows the distribution of times from division (of the mother cell) to the priming event for cells stemming from P and L cells. As expected, the former cells display positive times, as the decision is made after the last division; and the latter show negative times, as the decision is made in the mother (L) cell. Now, if cells are classified according to the division type they stem from, the results are slightly different (S9C Fig.). In this case, the distribution of times for AD divisions is basically the same as for P mother cells (cf. panels B and C) but the distribution for SD divisions includes a small tail upwards. This tail corresponds to the residual fraction of SD divisions that stem from a P cell rather than from an L cell. For a constant delay of the fluorescent reporter, the distributions of lag times between cell division and the reporter onset are basically the same but shifted (cf. panels D, C in S9 Fig.). Now, if we classify differentiated cells into those stemming from AD vs SD divisions, then the results are slightly different (S9C Fig.). Interestingly, even when the delay considered for the reporter is as long as 8 hours, the model predicts a very small fraction of cells from SD divisions that will turn on the reporter before dividing. We have observed this phenomenon in a few cells (Fig. 6 and S6 Table). The model simulations also anticipate a high degree of synchronization in the lag time of reporter onset between sibling cells (Figs. 5G and S9E).

We have also considered the effect t of introducing variability in the delay of the RFP onset. We assumed that the delay in the RFP onset is normally distributed around 8 hours with a standard deviation of 0.25 (i.e, that 99.99% of cells turn on the RFP between 7 and 9 hours after being primed, see S9F Fig.). This level of variability had little impact in the distribution of lag times in ACD and SCD originated cells (S9G Fig.) and also in the synchronization of sibling cells (S9H Fig.). As we increased the variability in the delay, we observed that synchronization progressively deteriorated.

Finally, we included the 11.6% false negative rate in the reporter (S4C Fig.) of the model. We did that by relabeling 11.6% of the SCD originated cells as being originated by ACD. This re-assignation of cells introduced a tail in the lower range of the distribution of lag times in ACD cells (S10 Fig.) but overall the results were not significantly affected.
